# Systematics of the Madagascar *Anelosimus* spiders: remarkable local richness and endemism, and dual colonization from the Americas

**DOI:** 10.3897/zookeys.509.8897

**Published:** 2015-06-22

**Authors:** Ingi Agnarsson, Brian B. Jencik, Giselle M. Veve, Sahondra Hanitriniaina, Diego Agostini, Seok Ping Goh, Jonathan Pruitt, Matjaž Kuntner

**Affiliations:** 1 Department of Biology, University of Vermont, Burlington, VT, USA; 2 Department of Entomology, National Museum of Natural History, Smithsonian Institution, Washington, DC, USA; 3 Department of Entomology, University of Madagascar, Antananarivo, Madagascar; 4 Department of Biology, University of Puerto Rico, Rio Piedras, Puerto Rico, USA; 5 Department of Biological Sciences, National University of Singapore, Singapore; 6 Department of Biological Sciences, University of Pittsburgh, PA, USA; 7 Institute of Biology, Scientific Research Centre, Slovenian Academy of Sciences and Arts, Ljubljana, Slovenia; 8 Centre for Behavioural Ecology and Evolution (CBEE), College of Life Sciences, Hubei University, Wuhan, Hubei, China

**Keywords:** Cobweb spiders, subsocial, Theridiidae, biogeography, colonization, radiation, congener coexistance

## Abstract

Despite the alarming rates of deforestation and forest fragmentation, Madagascar still harbors extraordinary biodiversity. However, in many arthropod groups, such as spiders, this biodiversity remains mostly unexplored and undescribed. The first subsocial Madagascan species of the theridiid spider genus *Anelosimus* were described in 2005 when six new species were found to coexist in the Périnet forest fragment within Andasibe-Mantadia NP. However, this discovery was based only on a few specimens and the extent of this Madagascan radiation has remained unknown. We here report on a thorough survey of >350 colonies from Périnet, and three pilot surveys into additional Madagascar forests (Ambohitantely, Ranamofana, and Montagne d’Ambre). The morphological, molecular and natural history data from these surveys facilitated a revised taxonomy and phylogenetic hypothesis of Madagascan *Anelosimus*. This subsocial clade currently comprises six previously known (*Anelosimus andasibe* Agnarsson & Kuntner, 2005, *Anelosimus may* Agnarsson, 2005, *Anelosimus nazariani* Agnarsson & Kuntner, 2005, *Anelosimus sallee* Agnarsson & Kuntner, 2005, *Anelosimus salut* Agnarsson & Kuntner, 2005, *Anelosimus vondrona* Agnarsson & Kuntner, 2005) and 10 new species: *Anelosimus ata* **sp. n.**, *Anelosimus buffoni* **sp. n.**, *Anelosimus darwini* **sp. n.**, *Anelosimus hookeri* **sp. n.**, *Anelosimus huxleyi* **sp. n.**, *Anelosimus lamarcki* **sp. n.**, *Anelosimus moramora* **sp. n.**, *Anelosimus tita* **sp. n.**, *Anelosimus torfi* **sp. n.**, *Anelosimus wallacei* **sp. n.**. With the exception of *Anelosimus may* and *Anelosimus vondrona*, all other species appear to be single forest endemics. While additional sampling is necessary, these data imply a much higher local richness and endemism in Madagascan forests than in any other comparable area globally. The phylogenetic results establish a sister clade relationship between the subsocial *Anelosimus* in Madagascar and the American ‘*eximius* group’, and between the solitary *Anelosimus decaryi* on Madagascar and a solitary American clade. These findings imply duplicate colonizations from America, an otherwise rare biogeographical pattern, calling for more detailed investigation of *Anelosimus* biogeography.

## Introduction

Madagascar is a biodiversity hotspot that has undergone extreme deforestation during the last century. What once was near continuous and vast forest along the eastern slopes of Madagascar is now fragmented, often into discontinuous patches. Many such isolated patches harbor unique diversity of species, with particularly well known vertebrate examples including endemic lemurs, chameleons, geckoes, snakes, frogs, and others (Agnarsson et al. 2014; Agnarsson and Kuntner 2012; Karanth et al. 2005; Raxworthy et al. 2007, 2008; Vences et al. 2003, 2004; Vidal et al. 2010; Yoder and Nowak 2006). However, for many groups of organisms, including notably diverse arthropod lineages such as hemipterans and beetles (Krishnankutty 2012; Rainio 2012), knowledge of species and their distribution in Madagascar remains very limited. Because such groups are critical for holistic understanding of the major events and factors—biotic and abiotic—responsible for diversification of lineages in Madagascar (Agnarsson et al. 2014; Agnarsson and Kuntner 2012; Dijkstra 2007; Fisher 1997) and other ancient islands (Gillespie and Roderick 2002), further taxonomic species discovery of arthropods in Madagascar is essential.

In spider research, Madagascar has been relatively neglected with the most notable exception being the four year inventory by the California Academy of Sciences under the leadership of Dr. Charles E. Griswold, and a few additional efforts (see the works of Agnarsson 2006; Agnarsson et al. 2010b; Alvarez-Padilla et al. 2012; Griswold 1997, 2000; Griswold and Ledford 2001; Jocqué 1994; Kuntner 2006; 2007; Kuntner and Agnarsson 2011; Wood et al. 2007). For example, Darwin’s bark spider exhibiting the web with the longest bridgelines and toughest silk of all spiders (Agnarsson et al. 2010a), was only discovered in the recent years (Kuntner and Agnarsson 2010), and despite lively recent research into their biology (Gregoric et al. 2011a-b), the Madagascar radiation of bark spiders remains largely undescribed (Gregorič et al. 2015).

Similarly, the first subsocial species of the spider genus *Anelosimus* Simon, 1891 were described from Madagascar only 10 years ago (Agnarsson and Kuntner 2005). This initial discovery of six related subsocial species in a single forest fragment (Périnet)—more than any other locality on Earth–indicated that Madagascar may be home to a rich radiation of these spiders. Here, we report on a thorough survey of Périnet, and three pilot surveys of additional forest patches, from the highly isolated fragments of Ambohitantely and Montagne d’Ambre National Parks, and from a National Park at the heart of the remaining continuous eastern slope forests, Ranamofana National Park. We describe the newly discovered species, offer basic information on their natural history, and place them in a global phylogenetic context.

## Methods

Spiders were collected in the field from Ambohitantely Special Reserve, Ankazobe district, 28.iv.2008 (Agnarsson and Kuntner), Montagne d’Ambre National Park, Antsiranana district, 4.iv.2008 (Agnarsson and Kuntner), Périnet Special Reserve, 3–20.iv.2008 and 13–26.xi.2008 (Agnarsson, Kuntner, Hanitriniaina and Rabarison), and Ranamofana National Park, Fianarantsoa district, 27.iv.–2.v.2013 (Suppl. material 1). Colonies were located in the forest along trails and rivers at tips of branches, often containing one or more dead leaves, which were webbed together as a retreat. Entire colonies were sampled into zip-lock bags and contents were subsequently sorted into sampling vials. In both Ambohitantely and Montagne d’Ambre sampling was opportunistic and rapid as only a few hours were available for sampling at each site. However, in Ranamofana basic observations were also made on the biology of the species, and in Perinét a systematic survey of >350 colonies was made.

Specimens were identified to species (Agnarsson and Kuntner 2005) and/or morphospecies under a Leica MZ16 dissecting microscope and photographed using the Visionary Digital BK lab system equipped with a Canon 5D camera and a 65mm macro 5x zoom lens. Multiple images taken at different focal planes were combined with Helicon Focus 5.3 and processed with Photoshop CS6 to adjust contrast and sharpness, and to create a white background. For photography, specimens were temporarily mounted in alcohol-based hand sanitizer (GermX containing 65% ethanol and no added perfumes or other chemicals), then covered with 75% ethanol. Most measurements were made directly from photographs using the ‘analyses’ tools in Photoshop, with additional measurements made, as needed, using a micrometer eye-piece in the Leica microscope. All measurements are in millimeters. Prosoma and abdomen length and height were measured in lateral view, the width in dorsal view, all measured at widest points. Leg segments were measured without the detachment of legs from the prosoma and are thus approximations. Female genitalia were excised using sharp forceps and digested using KOH for photography of internal genitalia. Photographs were assembled into plates and labeled in Illustrator CS6.

DNA was isolated from one to multiple individuals per putative morphospecies depending on specimen availability using the QIAGEN DNeasy Tissue Kit (Qiagen, Inc., Valencia, CA). We targeted fragments of two mitochondrial (cytochrome *c* oxidase subunit 1-COI, 16S rRNA) and two nuclear (Internal transcribed spacer-ITS2 and 28S rRNA) loci previously demonstrated to be effective phylogenetic markers at low taxonomic levels for spiders (Agnarsson et al. 2007, 2010b, 2013). We amplified COI with the LCO1490 (Folmer et al. 1994), and C1-N-2776 primers (Hedin and Maddison 2001). We used 16SA and B primers (Simon et al. 1994) to amplify 16S, the ITS-5.8S (FITS) and ITS-28S (RITS, or ITS 4) primers for ITS2 (White et al. 1990), and 28Sc and 28So primers for 28S (Whiting et al. 1997). Sequencing was done at the University of Arizona. Sequences were submitted to GenBank (accession numbers: KR909226–KR909300, KT174673–KT175005).

Chromatograms were interpreted employing Phred and Phrap (Green 2009; Green and Ewing 2002) through the Chromaseq 1.01 module (Maddison and Maddison 2010) in the evolutionary analysis program Mesquite 2.75 (Maddison and Maddison 2014) with default parameters. The sequences were then proofread by examining chromatograms by eye. The data were aligned in MAFFT (Katoh 2013) through the online portal EMBL-EBI, using default settings and increasing the tree rebuilding and maxiterate settings to 100. Gaps were treated as missing data. For Bayesian analyses, the appropriate substitution model was selected with jModelTest 2.1.4 (Posada 2008) using the AIC criterion to select among the 24 models implemented in MrBayes (Huelsenbeck and Ronquist 2001). The best model for 28S, COI and 16S was GTR+G+I, for ITS2 was GTR+G, and for previously published ND1 sequences HYK+G. We ran Bayesian analysis of the five loci concatenated in MrBayes V3.2.2 (Ronquist and Huelsenbeck 2003) through the CIPRES online portal. The concatenated analysis was partitioned by locus. We ran the Markov chain Monte Carlo with four chains for 10,000,000 generations, sampling the Markov chain every 1,000 generations. The results were examined in Tracer 1.5 (Rambaut and Drummond 2007) to verify proper mixing of chains and that stationarity had been reached, and to determine adequate ‘‘burnin’’. Trees were edited in Mesquite and then exported as PDF files and all figures were compiled and finalized in Adobe Illustrator.

Species were delimited based on a combination of molecular and morphological diagnosability. Specimens from different areas showing identical or near identical DNA barcoding (COI) haplotypes and sharing detailed genital morphology were treated as conspecific. Specimens differing distinctly in DNA barcodes, generally over 3% sequence divergence (e.g. Hebert et al. 2004) but in some cases as little as 2% (2% is close to minimal distance between closely related previously described *Anelosimus* species pairs, e.g. Agnarsson 2007, 2012a, b) from other individuals in the analysis, and showing genitalic differences, albeit subtle, were treated as heterospecific. Paucity of specimens of some species prevented establishing a ‘barcoding gap’ (e.g. Čandek and Kuntner 2015) but for the most closely related species that have multiple specimens available all are separated by a gap of >10 times greater inter- than intraspecific genetic distances (Table 1). All types are deposited in the National Museum of Natural History, Smithsonian Institution (NMNH).

**Table 1.** Estimates of divergence (Genetic distances) within and among species based on averaging substitutions per site over all sequence pairs under a Maximum Likelihood model with gamma distributed rate variation. The analyses were done including 1011 COI positions for 324 individuals in MEGA6 (Tamura et al. 2013).

		Intraspecific	1	2	3	4	5	6	7	8	9	10	11	12	13	14	15	16
1	*Anelosimus moramora*	n/a	X															
2	*Anelosimus hookeri*	n/a	n/a	X														
3	*Anelosimus lamarcki*	0.002	0.111	0.032	X													
4	*Anelosimus tita*	n/a	0.149	0.060	0.053	X												
5	*Anelosimus torfi*	n/a	0.162	0.051	0.076	0.078	X											
6	*Anelosimus andasibe*	0.000	0.118	0.038	0.022	0.054	0.080	X										
7	*Anelosimus wallacei*	0.002	0.113	0.028	0.022	0.055	0.070	0.023	X									
8	*Anelosimus buffoni*	0.001	0.119	0.029	0.023	0.048	0.074	0.021	0.019	X								
9	*Anelosimus ata*	0.002	0.129	0.055	0.058	0.056	0.076	0.056	0.055	0.052	X							
10	*Anelosimus darwini*	0.000	0.128	0.055	0.055	0.057	0.059	0.053	0.048	0.046	0.052	X						
11	*Anelosimus huxleyi*	0.010	0.123	0.043	0.049	0.047	0.078	0.053	0.046	0.045	0.055	0.056	X					
12	*Anelosimus may*	0.010	0.123	0.065	0.063	0.066	0.076	0.067	0.057	0.057	0.051	0.045	0.063	X				
13	*Anelosimus nazariani*	0.001	0.131	0.071	0.066	0.054	0.075	0.065	0.062	0.062	0.059	0.054	0.063	0.059	X			
14	*Anelosimus sallee*	0.001	0.123	0.062	0.057	0.056	0.079	0.057	0.055	0.051	0.060	0.058	0.051	0.060	0.064	X		
15	*Anelosimus salut*	0.001	0.154	0.082	0.080	0.075	0.052	0.082	0.080	0.075	0.091	0.072	0.083	0.092	0.088	0.089	X	
16	*Anelosimus vondrona*	0.000	0.103	0.022	0.033	0.056	0.075	0.041	0.035	0.035	0.062	0.058	0.051	0.068	0.064	0.059	0.083	X
	average	0.003																

In addition to describing new species, we also redescribe known species to include first descriptions of males, or to correct previous taxonomic errors, and include illustrations and DNA barcodes of all Madagascan *Anelosimus* to facilitate future identification from a single source.

## Results and discussion

**Figure 1.**
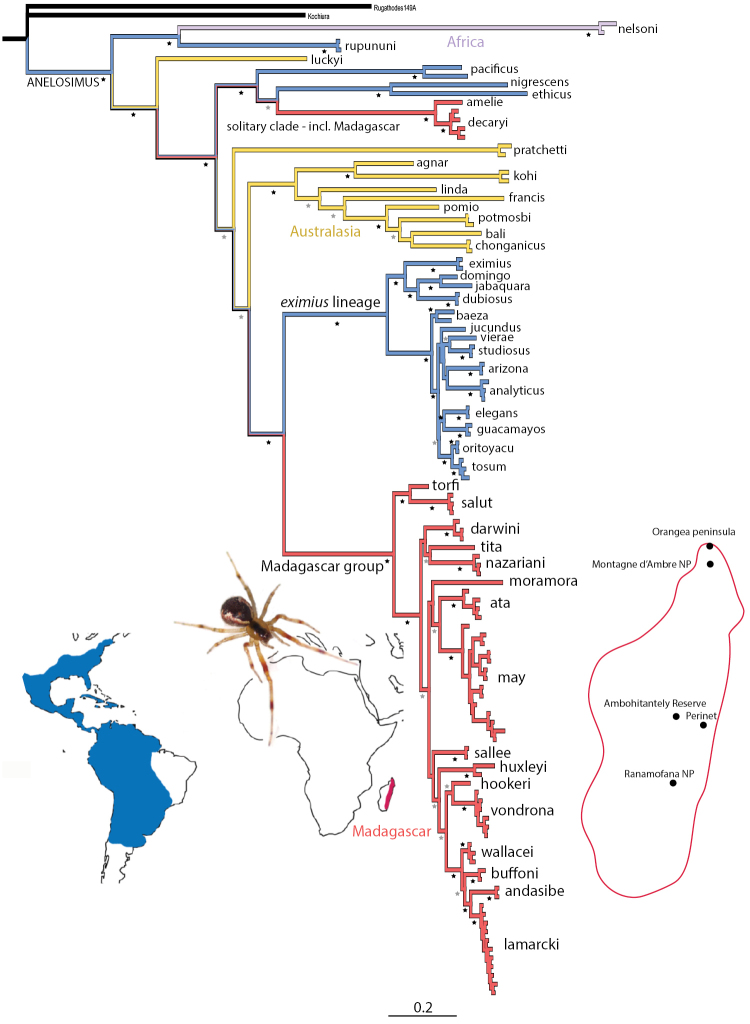
A Bayesian phylogeny of Madagascar *Anelosimus* and worldwide relatives. Terminal taxa are replaced by species names, full results including all taxon details are found in Suppl. material 1. Black stars indicate 100 posterior probability support, gray stars 85–99%. The replicated sister relationship between Madagascan and American clades is highlighted in blue and red, with the total distribution of each lineage indicated on the maps in lower left. Dots on right map indicate collection localities for the current study. Spider photograph is of the European *Anelosimus vittatus*, adapted from a photo by Glenn Halvor Morka.

Our total dataset contained specimens from >400 colonies and sequence data from 357 Madagascan individuals, plus global representatives. COI barcodes, totaling 1011 bp, were obtained from most sequenced specimens and 336 individuals were included in the barcode analysis (Supplementary Material). Three additional loci were obtained from a small subset for a phylogenetic matrix of 3409 bp for 114 global terminals (Figure 1). Morphological examination and phylogenetic analyses reveal ten new *Anelosimus* species from four forest fragments in Madagascar. The only intensely surveyed forest, Périnet Special Reserve in the Andasibe-Mantadia National Park contained a remarkable assemblage of 10 species, adding four new species to the six recently described ones. Some of the species are fully allopatric with respect to their closest relative in the tree, consistent with allopatric speciation in this clade. However, others occur in sympatry with their closest relatives. *Anelosimus* is globally distributed, but this local diversity far exceeds that of any other locality in the World. Other forests in Madagascar contained fewer species, but in two of them we still uncovered impressive diversity given the limited sampling. For example, we were able to collect for only a few hours in Ambohitantely, finding approximately 15 colonies, and these contained a total of 5 species, thereof four new to science. Further systematic sampling of these and other montane forest fragments in Madagascar thus promises to continue to discover diversity in this clade. *Anelosimus* has been most thoroughly studied in the Americas (Agnarsson et al. 2007; 2010c; Albo et al. 2007; Aviles 1986; Aviles and Bukowski 2006; Aviles and Gelsey 1998; Aviles et al. 2006) where the genus was thought to be most species rich. However, the island of Madagascar now seems to harbor a comparable radiation.

Curiously, our phylogenetic results establish that the subsocial montane forest ‘Madagascar group’ is sister to the most diverse American group, the ‘*eximius* lineage’, and the solitary group from Madagascar is sister to solitary species also from the Americas (Fig. 1). These results do not support prior hypothesis of the sister relationship of Madagascar and African *Anelosimus* (Agnarsson et al. 2010b). Given that the genus is likely much too young to reflect a Gondwanan distribution, somewhere between 15–35 my (Agnarsson et al. unpublished data), this close relatedness of Madagascar to American species is difficult to explain. The genus is likely diverse—though poorly studied—in Africa, however, species studied to date seem unrelated to either of the two Madagascar clades. These are the South African *Anelosimus nelsoni* Bryant, 1945 included here, and a diverse clade of mostly undescribed east African species that have been phylogenetically placed in a diverse African plus Asian clade, based on morphological data (Agnarsson 2006). The majority of the biota of Madagascar traces back its history to colonization events from Africa with only about 5% of examined groups colonizing the island from the Americas (Agnarsson and Kuntner 2012). Seeing such a rare pattern replicated twice within a single genus is thus a significant finding. Further systematic sampling of African *Anelosimus* is necessary, however, to adequately address the biogeography of the lineage.

The intense survey of Périnet secured fresh samples of all known Madagascan *Anelosimus* species and led to the discovery of males of several species. These species are all re-illustrated and males described. Several new species were also discovered in Périnet and in pilot surveys of Ambohitantely, Ranamofana, and Montagne d´Ambre. Many of these new species are only known from single or a few female specimens, which is far from ideal for taxonomic purposes. These are nevertheless described here to highlight the diversity of this group in Madagascar and especially to call attention to the uniqueness of the tiny and isolated forest fragment of Ambohitantely. Our findings underline the need to thoroughly survey eastern Madagascan montane forests, and to preserve their extraordinary biodiversity.

*DNA delimitation and diagnosis*. Given that it can be difficult to delimit and distinguish species of Madagascan *Anelosimus* based on morphology alone, especially in species where males are unknown, we offer DNA diagnosis in addition to standard morphological diagnosis. In total we obtained 334 COI barcodes for specimens of the 16 species. Average distances among species barcodes ranged from 16.2% to 1.9% (Table 1). One clade of four species (*Anelosimus buffoni* sp. n., *Anelosimus wallacei* sp. n., *Anelosimus lamarcki* sp. n. and *Anelosimus andasibe*) has the lowest average interspecific distances ranging from 1.9–2.2%. However, there are several lines of evidence suggesting these are heterospecific. All are represented by multiple individuals and show intraspecific distances ranging from 0.1–0.2% – roughly 10 times less than that among species, often referred to as a ‘barcoding gap’ (Table 1). Given that three of these coexist, not only regionally but can be found at trivial distances in colonies sharing the same trees and branches we do not expect these genetic distances to reflect any dispersal biases, such as male-mediated dispersal. Furthermore, even the most densely sampled species within Périnet with nearly 100 COI sequences and those found in more than one of the forests sampled, have low to very low intraspecific sequence divergences suggesting panmixia at local and even regional scales. Finally, distances of around 2% separate some closely related *Anelosimus* species pairs elsewhere, and even lower distances are known to separate sympatric species of other organisms, such as skippers in Guanacaste (Hebert et al. 2004).

## Taxonomy

Species diagnoses are based on the standard DNA COI barcodes with a reference alignment starting at base 1 of the routinely used 658 bp fragment and in the matrix submitted as supplementary material (and available from the authors) but extending beyond the standard barcode to cover a 1000 bp fragment. We list 1) unique mtDNA nucleotide substitutions at alignment positions for each species as distinct diagnostic features, e.g. G (100) indicating this species uniquely has a G in position 100, following e.g. Bond and Stockman (2008); 2) and also unique combination of nucleotide substitutions that may be shared with one or maximally two additional species, e.g. G (184, except *Anelosimus buffoni* sp. n. and *Anelosimus andasibe*) indicating this species can be diagnosed from all species except those named in parenthesis by this substitution, and from all species by the combination of multiple such partially shared substitutions. This approach does not exhaust the diagnosability of species based on DNA barcodes. For example, four species (*Anelosimus may*, *Anelosimus torfi*, *Anelosimus darwini*, and *Anelosimus wallacei*), none of which are sister species, can each be diagnosed from their respective closest relatives by A (241). In fact A (241) is one of relatively few features that readily diagnoses *Anelosimus wallacei* from the related and very similar *Anelosimus buffoni*. However, listing every DNA substitution-based diagnosis is not feasible and we set the arbitrary limit to substitutions shared with no more than two species in our diagnosis sections. The matrix of barcodes is made available as supplementary material for complete diagnoses. Four of the 16 species were represented only by a single sequence such that no variation is known and the diagnoses involving these species are thus preliminary – a common enough predicament with morphological diagnoses in rare species. However, given that the average within species distances among densely sampled species were 0.3% and 0.1% for those species limited to a single forest, there is no a priory reason to expect these rarely collected species to be outliers with abundant intraspecific variation.

### Family Theridiidae Sundevall, 1833

#### Genus Anelosimus

Taxon classificationAnimaliaAraneaeTheridiidae

Simon, 1891

##### Type species.

*Anelosimus socialis* Simon, 1891 = *Anelosimus eximius* (Keyserling, 1884).

#### The “Madagascar group” sensu Agnarsson and Kuntner (2005) and Agnarsson (2006)

**Figure 2.**
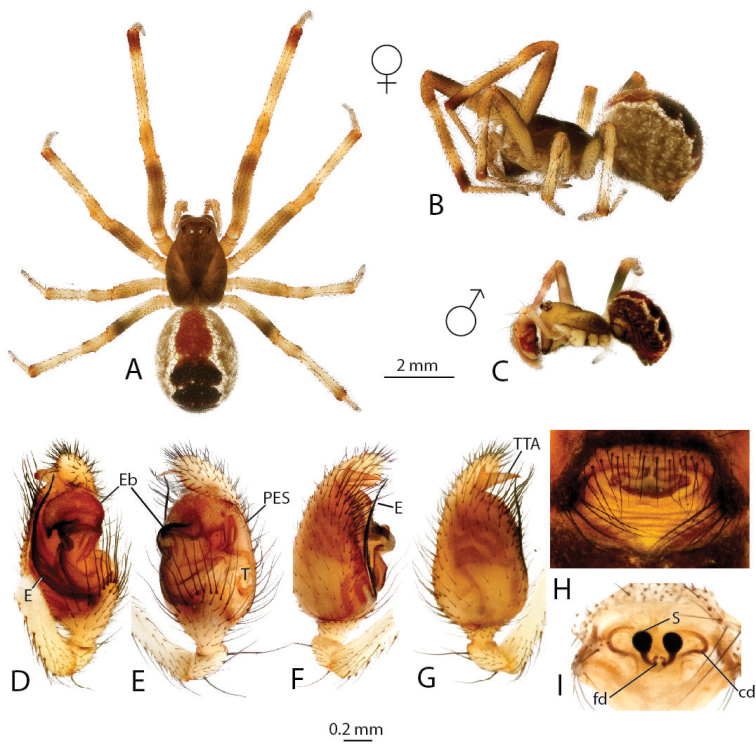
*Anelosimus may*: **A–B** female dorsal and lateral views **C** male lateral view **D–G** male palp ventral, subectal, mesal, dorsal **H** epigynum ventral **I** epigynum dorsal.

**Figure 3.**
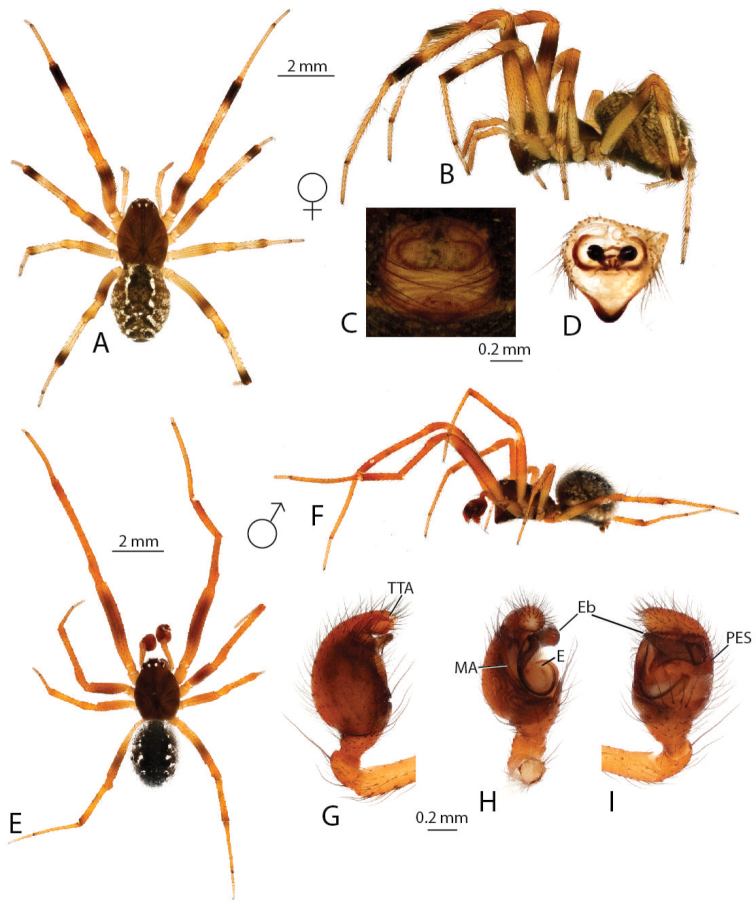
*Anelosimus vondrona*: **A–B** female dorsal, lateral **C** epigynum ventral view **D** epigynum, dorsal **E–F** male dorsal, lateral **G–I** male palp mesal, ectal, ventral.

Brief diagnosis (see Agnarsson and Kuntner (2005) for a full diagnosis): Males uniquely with a large and distally rugose embolic division b (Figs 2D–E, 3H–I) and a hooked proximal embolic sclerite (PES, Fig. 2E) in between the tegulum and the embolus. Sperm duct pathway of male palps highly complex with up to ten switchbacks. Females uniquely possess a pendulum-like epigynal septum (Figs 2H, 3C).

##### Anelosimus may

Taxon classificationAnimaliaAraneaeTheridiidae

Agnarsson, 2005 in Agnarsson and Kuntner (2005)

###### Notes.

The species is here redescribed to clarify earlier taxonomic confusion; the female previously described (see Agnarsson and Kuntner 2005), was in fact not conspecific with the male holotype, and is below described as *Anelosimus ata* sp. n. Here we therefore redescribe the female of *Anelosimus may* based on specimens collected at the type locality of the male. An additional male specimen also allowed a more detailed documentation of the male palp (Fig. 2D–G).

###### Type material.

**Holotype** male from Ambohitantely Special Reserve, Analamanga region, Ankazobe district, Madagascar, (18.161°S, 47.302°E), 17–22.iv.2001, montane forest, 1500 m alt, col. J. J. Rafanomezantsoa et al., in CAS, examined (see Agnarsson and Kuntner 2005).

###### Other material.

Additional three females from same locality, 28.iv.2008, col. Agnarsson and Kuntner, a male and multiple females from Périnet Special Reserve, Andasibe Mantadia National Park, Madagascar (18.933°S, 48.417°E), montane forest, 900–1000 m alt, 3–20.iv.2008 and 12–28.xi.2008, col. Agnarsson, Kuntner, and Hanitriniaina and eight females from Ranamofana National Park (21.25°S, 47.43°E), montane rainforest, 980–1050 m alt, 27.iv.–2.v.2013, col. Pruitt.

###### Diagnosis.

Males are diagnosed from other species by the shape of the theridiid tegular apophysis, bifurcated with the lower branch longer than the upper (Fig. 2G) and the voluminous Eb (Fig. 2E). Females differ from others of the Madagascar group, except *Anelosimus ata* sp. n. by the anchor-shaped septum (Fig. 2H) and from *Anelosimus ata* sp. n. by the more acute curving of the copulatory duct (Fig. 2I). *Anelosimus may* can be diagnosed from other Madagascan *Anelosimus* on the basis of the following unique mtDNA nucleotide substitutions at the following standard DNA barcode alignment positions: A (31), A (223), A (274), G (517), G (529). It can also be readily diagnosed from most other *Anelosimus* based the following partially shared nucleotide substitutions, and all other species by their unique combination: T (58, except *Anelosimus huxleyi* sp. n.), G (100, except *Anelosimus hookeri* sp. n., and some *Anelosimus darwini* sp. n.), T (181, except *Anelosimus ata* sp. n.), G (244, except *Anelosimus darwini* sp. n.), T (352, except *Anelosimus sallee* and *Anelosimus darwini* sp. n.), T (355, except *Anelosimus ata* sp. n.), T (484, except *Anelosimus torfi* sp. n. and *Anelosimus nazariani*), T (781, except *Anelosimus huxleyi* sp. n. and *Anelosimus salut* sp. n.), G (805, except rarely *Anelosimus nazariani*), A (871, except *Anelosimus nazariani*), G (973, except *Anelosimus sallee*).

###### Description.

*Female*: Total length 6.02 Cephalothorax 2.77 long, 1.94 wide, 1.58 high, brown. Sternum 1.49 long, 1.23 wide, extending half way between coxae IV, brown. Abdomen 3.85 long, 2.74 wide, 2.8 high. Brown base with white line and dot patterns with red near the spinnerets. Eyes subequal in size about 0.14 in diameter. Clypeus height about 2.9 times one AME diameter. Chelicerae with one large tooth, three denticles prolaterally. Leg I femur 3.4, patella 1.06, tibia 3.22, metatarsus 2.92, tarsus 1.18. Leg formula 2314, with leg 4 slightly longer than leg 1. Legs 1 and 2 brown, legs 3 and 4 light brown-yellow with dark brown at junctions between tibia and metatarsus, and metatarsus and tarsus. 4 small trichobothria dorsally on tibia I, 4 on tibia II. Trichobothria on all metatarsi (1–2), 4–5 dorsal trichobothria on female palpal tibia.

*Variation*: Total length 5.70–6.20, Cephalothorax 2.60–2.80, femur 1 3.00–3.50.

*Male* (from Ranamofana, see Agnarsson and Kuntner (2005) for description of holotype male): Total length 4.01. Cephalothorax 2.05 long, 1.61 wide, 0.91 high, dark brown. Abdomen 2.21 long, 1.54 wide, 1.41 high. Light brown base with black/brown spots, two jagged white longitudinal stripes and a central red longitudinal band. Eyes subequal in size about 0.13 in diameter. Leg I femur 3.15, patella 0.76, tibia 2.98, metatarsus 2.65, tarsus 1.05. Leg formula 1243. Leg yellow, with alternating light and dark reddish shaded bands.

*Variation*: Total length 3.25–4.01, Cephalothorax 1.63–2.05, femur I 2.67–3.15.

###### Distribution.

Eastern Madagascan montane forest. This is the most widespread species of the Madagascar group, documented from Périnet, Ambohitantely and Ranamofana, and can be expected to be found in additional montane forest reserves in eastern Madagascar.

###### Natural history.

In Ranamofana, eight complete *Anelosimus may* colonies along trails in the forest interior were found. We found two colonies containing one adult female and her egg case and eight colonies containing a female with a group of small juveniles, likely instars I–III post egg sac. Females actively guarded their egg cases by seizing them in their chelicerae. We also noted one instance of a female feeding her young via regurgitation. Our observations indicate that *Anelosimus may* primarily exhibits subsocial behavior, as do other members of the Madagascar group. An unidentified salticid inhabited six of the eight colonies sampled.

##### Anelosimus vondrona

Taxon classificationAnimaliaAraneaeTheridiidae

Agnarsson & Kuntner, 2005

Fig. 3

###### Notes.

We here describe the male of *Anelosimus vondrona* for the first time and illustrate both sexes to facilitate identification.

###### Type material.

**Holotype** female from Périnet Special Reserve (P.N. Andasibe Mantadia), Toamasina Province, Madagascar, (18.935°S, 48.418°E), 7–8.v.2001, montane forest, 900–1000 m, (I. Agnarsson and M. Kuntner), in NMNH, examined.

###### Other material.

Multiple additional specimens from same locality, 3–20.iv.2008 and 12–28.xi.2008, col. Agnarsson, Kuntner, and Hanitriniaina, and from Ranamofana National Park (21.25°S, 47.43°E), montane rainforest, 980–1050 m alt, 27.iv. – 2.v.2013, col. Pruitt.

###### Diagnosis.

*Anelosimus vondrona* females can be diagnosed from all other species except *Anelosimus huxleyi* by the relatively broad septum that extends the entire width of the epigynum (Fig. 3C) and from *Anelosimus huxleyi* by the less heavily sclerotized lower margin of the epigynal plate. Males can be diagnosed by the shape of the TTA with curved and elongate upper branch (Fig. 3G), and the shape of the Eb (Fig. 3H–I). *Anelosimus vondrona* can be diagnosed from other Madagascan *Anelosimus* on the basis of the following unique mtDNA nucleotide substitutions at the following standard DNA barcode alignment positions: G (802), T (820). It can also be readily diagnosed from most other *Anelosimus* based the following partially shared nucleotide substitutions, and all other species by their unique combination: A (163, rarely also in *Anelosimus nazariani*), G (466, except *Anelosimus hookeri* sp. n.), G (493, except some *Anelosimus may*), G (521, except *Anelosimus salut*), G (619, except *Anelosimus huxleyi* sp. n.), G (628, except some *Anelosimus may*), G (655, except some *Anelosimus huxleyi* sp. n.), G (760, except most *Anelosimus nazariani*), G (799, except *Anelosimus buffoni* sp. n.)

###### Description.

*Male* (same locality as holotype): Total length 4.47 Cephalothorax 2.03 long, 1.53 wide, 0.49 high. Sternum 1.11 long, 0.94 wide, extending halfway between coxae IV, dark brown. Abdomen 2.40 long, 1.87 wide, 1.89 high, color (Fig. 3A). Eyes subequal in size about 0.13 in diameter. Clypeus height about 2 times one AME diameter Chelicerae with one large tooth, and 3–4 denticles retrolaterally Leg 1 femur 3.16, patella 0.88, tibia 3.15, metatarsus 2.60, tarsus 1.11. Leg formula 1243 Legs are light brown-yellow. 7 small trichobothria dorsally on tibia I and II, and two dorsally on metatarsi.

###### Distribution.

Eastern Madagascan montane forest, documented from Périnet and Ranamofana.

###### Natural history.

In Ranamofana, we sampled ten colonies of *Anelosimus vondrona*. Six colonies contained a singleton female and four colonies contained a singleton female with a discolored, collapsed egg sac. All of these colonies were found along roadsides and ornamental shrubbery. In Périnet a large number of colonies were collected, almost exclusively in open forest, including many with female and up to 53 spiderlings coexisting. An adult female was more commonly present in webs with small juveniles but also found in some nests containing antepenultimate and subadult (5^th^-6^th^ instar) juveniles, suggesting prolonged cohabitation of mother and young. Foreign spiders were abundant in *Anelosimus vondrona* colonies, including several saliticids, a sparassid, a thomisid, and many theridiids.

##### Anelosimus salut

Taxon classificationAnimaliaAraneaeTheridiidae

Agnarsson & Kuntner, 2005

Fig. 4

###### Notes.

New material of males of this species allowed a more detailed study of the palpal organ and we provide new illustrations and diagnosis of the male; the original description included a single drawing (see Agnarsson and Kuntner 2005, fig. 5D).

###### Type material.

**Holotype** male and paratype female, Périnet Special Reserve (P.N. Andasibe Mantadia), Toamasina Province, Madagascar, (18.935°S, 48.418°E), 24.xii.2001, montane forest, 1000 m, col. M. E. Irwin, E. I. Schlinger, H.H. Rasolondalao, in CAS, examined.

###### Other material.

Additional specimens from same locality, 3–20.iv.2008 and 12–28.xi.2008, col. Agnarsson, Kuntner, and Hanitriniaina.

###### Diagnosis.

**Figure 4.**
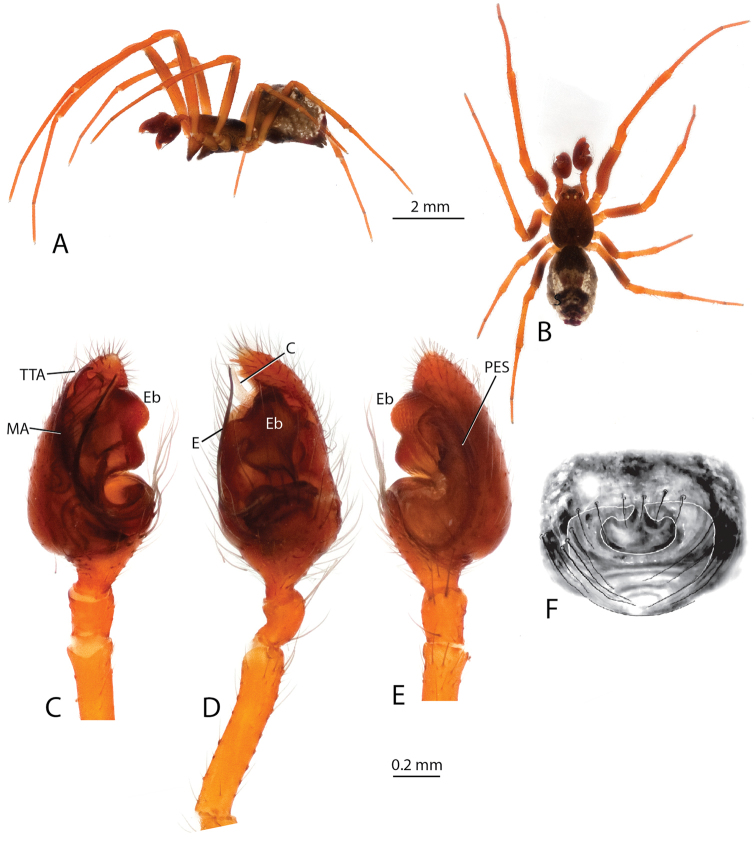
*Anelosimus salut*: **A–B** male lateral and dorsal views **C–E** male palp mesal, ectal, ventral **F** epigynum, ventral.

**Figure 5.**
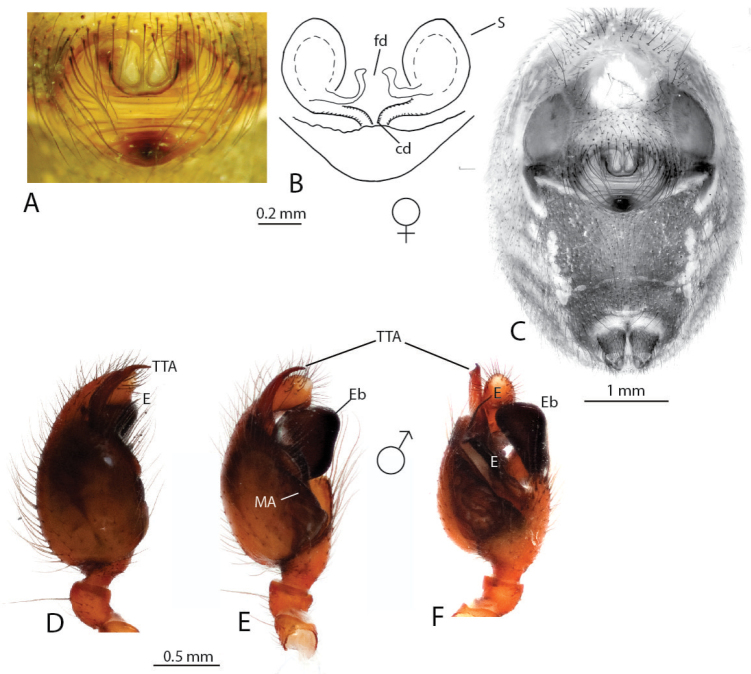
*Anelosimus nazariani*: **A** epigynum, ventral **B** epigynum, dorsal; C, female abdomen, ventral **D–F** male palp dorsal, mesal, ventral.

*Anelosimus salut* females can be diagnosed by having a broad ‘inverted T-shape’ septum that differs from *Anelosimus vondrona* in not extending the entire length of the epigynum (Fig. 5J). Males can be diagnosed from all other *Anelosimus* by the relatively short bifurcated TTA (Fig. 4C) and the bilobed embolic division b that is longer and narrower than in other species (Fig. 4D). *Anelosimus salut* can be diagnosed from other Madagascan *Anelosimus* on the basis of the following unique mtDNA nucleotide substitutions at the following standard DNA barcode alignment positions: A (38), T(43), T(97), T (369), A (371), T (415), G (460), A (470), A (494), A (568), T (796). It can also be readily diagnosed from most other *Anelosimus* based the following partially shared nucleotide substitutions, and all other species by their unique combination: A (256, except *Anelosimus torfi* sp. n. and *Anelosimus hookeri* sp. n.), T (370, except *Anelosimus torfi* sp. n.), T (412, except *Anelosimus torfi* sp. n.), A (469, except *Anelosimus torfi* sp. n.). A (474, except *Anelosimus nazariani*), G (521, except *Anelosimus vondrona*), G (541, except *Anelosimus sallee* and some *Anelosimus wallacei* sp. n.), A (622, except *Anelosimus torfi* sp. n.), T (631, except *Anelosimus darwini* sp. n.), A (754, except *Anelosimus torfi* sp. n.), T (781, except *Anelosimus may* and *Anelosimus huxleyi* sp. n.), T (940, except *Anelosimus torfi* sp. n.), A (961, except *Anelosimus torfi* sp. n.), G (994, except most *Anelosimus huxleyi* sp. n.).

###### Distribution.

Only known from type locality.

###### Natural history.

As other species in this group *Anelosimus salut* appears to be subsocial with colonies consisting of single females and up to 39 spiderlings.

##### Anelosimus nazariani

Taxon classificationAnimaliaAraneaeTheridiidae

Agnarsson & Kuntner, 2005

Fig. 5

###### Notes.

The male of *Anelosimus nazariani* is here described and diagnosed for the first time, the female epigynum is re-illustrated.

###### Type material.

**Holotype** and paratype females from Périnet Special Reserve (P.N. Andasibe Mantadia), Toamasina Province, Madagascar, (18.935°S, 48.418°E), 7–8.v.2001, montane forest, 900–1000 m, (I. Agnarsson and M. Kuntner), in NMNH, examined.

###### Other material.

Additional specimens from same locality, 3–20.iv.2008 and 12–28.xi.2008, col. Agnarsson, Kuntner, and Hanitriniaina.

###### Diagnosis.

*Anelosimus nazariani* differs from other species in being distinctly the largest *Anelosimus* species recorded to date with female total length exceeding 7 mm, with other species ranging from 1.9–5.5 mm. The males are easily diagnosed by the dark, bulky, and comparatively smooth Eb (Fig. 5E–F), and all but *Anelosimus sallee* by the very elongated upper branch of the TTA (Fig. 5D–E). The TTA differs in shape from that of *Anelosimus sallee*, being less curved. The epigynum differs from all but *Anelosimus andasibe*, *Anelosimus buffoni* sp. n., and *Anelosimus wallacei* sp. n. by the W pattern on the septum, and from these three by the larger distance between the septum and the epigynal margin (Fig. 5A). *Anelosimus nazariani* can be diagnosed from other Madagascan *Anelosimus* on the basis of the following unique mtDNA nucleotide substitutions at the following standard DNA barcode alignment positions: T (24), T(45), T(100), T (202), G (322), T (424), G (583), T (814), T (859). It can also be readily diagnosed from most other *Anelosimus* based the following partially shared nucleotide substitutions, and all other species by their unique combination: A (46, except *Anelosimus torfi* sp. n.), T (121, except *Anelosimus tita* sp. n.), T (127, except *Anelosimus darwini* sp. n. and *Anelosimus ata* sp. n.), T (130, except *Anelosimus darwini* sp. n.), G (262, except *Anelosimus tita* sp. n.), G (307, except some *Anelosimus salut*), G (313, except *Anelosimus sallee* and some *Anelosimus huxleyi* sp. n.), A (474, except *Anelosimus salut*), T (479, except *Anelosimus andasibe*), T (484, except *Anelosimus may* and *Anelosimus torfi* sp. n.), G (556, except *Anelosimus darwini* sp. n.), G (736, except some *Anelosimus may*), G (745, expect *Anelosimus wallacei* sp. n.and some *Anelosimus ata* sp. n.), G (841, except *Anelosimus torfi* sp. n.), A (871, except *Anelosimus may*).

###### Description.

*Male*: Total length 5.89 Cephalothorax 2.70 long, 1.99 wide, 0.27 high. Sternum 1.40 long, 1.17 wide, extending halfway between coxae IV, light brown. Abdomen 3.19 long, 2.31 wide, 2.08 high (add color). Eyes subequal in size about 0.15 in diameter. Clypeus height about times one AME diameter Chelicerae with one large tooth, 4–5 denticles retrolaterally Leg 1 femur 3.71, patella 1.18, tibia 3.94, metatarsus 3.45, tarsus 1.23 Leg formula 1243 Legs light brown-yellow with brown at junctions between tibia and metatarsus, and metatarsus and tarsus. 7 small trichobothria dorsally on tibia I and II, 3 dorsally on metatarsi.

###### Distribution.

Only known from type locality.

###### Natural history.

As in other species of this group a female can be found in its web with close to 50 juveniles and juveniles appear to cohabit in the web until close to adulthood.

##### Anelosimus sallee

Taxon classificationAnimaliaAraneaeTheridiidae

Agnarsson & Kuntner, 2005

Fig. 6

###### Notes.

The species is rediagnosed and genitalia re-illustrated.

###### Type material.

**Holotype** male, paratype female from Périnet Special Reserve (P.N. Andasibe Mantadia), Toamasina Province, Madagascar, (18.935°S, 48.418°E), 24.xii.1999 (M.E. Irwin et al.), in CAS, examined.

###### Other material.

Additional specimens from same locality, 3–20.iv.2008 and 12–28.xi.2008, col. Agnarsson, Kuntner, and Hanitriniaina.

###### Diagnosis.

**Figure 6.**
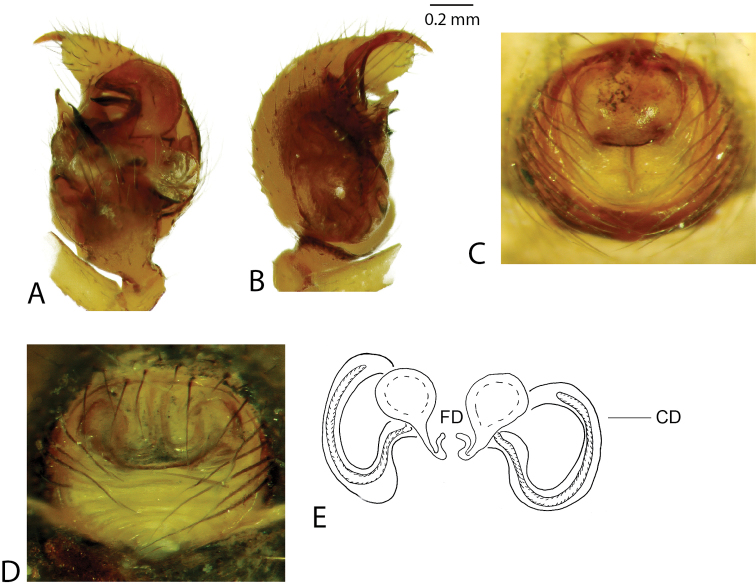
*Anelosimus sallee*: **A–B** palp, ventral, mesal **C** epigynum, ventral. *Anelosimus andasibe*: **D–E** epigynum, ventral, dorsal.

Males are readily diagnosed from all species other than *Anelosimus nazariani* by the elongate upper branch of the TTA (Fig. 6B) and from *Anelosimus nazariani* by the greater curvature of this branch. Females can be diagnosed by the shape of the septum being almost as high as wide (Fig. 5C). *Anelosimus sallee* can be diagnosed from other Madagascan *Anelosimus* on the basis of the following unique mtDNA nucleotide substitutions at the following standard DNA barcode alignment positions: G (190), C (284), C (401), G (403), A (421), G (433), A (482), A (718). It can also be readily diagnosed from most other *Anelosimus* based the following partially shared nucleotide substitutions, and all other species by their unique combination: G (211, except some *Anelosimus may*), G (313, except *Anelosimus nazariani* and some *Anelosimus huxleyi* sp. n.), T (139, except *Anelosimus huxleyi* sp. n.), T (352, except *Anelosimus may* and *Anelosimus darwini* sp. n.), G (541, except *Anelosimus salut* and some *Anelosimus wallacei* sp. n.), G (550, except rarely *Anelosimus nazariani*), T (838, except *Anelosimus huxleyi* sp. n.), G (934, except some *Anelosimus nazariani*), G (973, except *Anelosimus may*).

###### Distribution.

Only known from type locality.

###### Natural history.

This species is rare at the type locality, and too few colonies have been sampled to comment on its natural history, though it is expected to be subsocial like related species.

##### Anelosimus andasibe

Taxon classificationAnimaliaAraneaeTheridiidae

Agnarsson & Kuntner, 2005

Fig. 6E–F

###### Notes.

The species, known only from females, is rediagnosed and genitalia re-illustrated.

###### Type material.

**Holotype** female from Périnet Special Reserve (P.N. Andasibe Mantadia), Toamasina Province, Madagascar, (18.935°S, 48.418°E), 7–8.v.2001, montane forest, 900–1000 m, (I. Agnarsson and M. Kuntner), in NMNH, examined.

###### Other material.

Additional specimens from same locality, 3–20.iv.2008 and 12–28.xi.2008, col. Agnarsson, Kuntner, and Hanitriniaina.

###### Diagnosis.

*Anelosimus andasibe* differs from all but *Anelosimus nazariani*, *Anelosimus buffoni* sp. n., and *Anelosimus wallacei* sp. n. by the W-shaped septum (Fig. 6E), and from *Anelosimus nazariani* by the small distance between the septum and the epigynal margin and by being smaller. Clear diagnostic features separating females of the very similar *Anelosimus andasibe*, *Anelosimus buffoni* sp. n., and *Anelosimus wallacei* sp. n. have not been established, however, we predict they will be readily diagnosable based on palpal organs once males are discovered. *Anelosimus andasibe* can be diagnosed from *Anelosimus wallacei* sp. n. by lacking substitution A (241), and from *Anelosimus wallacei* sp. n. and *Anelosimus buffoni* sp. n. by lacking substitution G (249). It can be diagnosed from other Madagascan *Anelosimus* on the basis of the following unique mtDNA nucleotide substitutions at the following standard DNA barcode alignment positions: C (124), G (415), G (496), G (769). It can also be readily diagnosed from most other *Anelosimus* based the following partially shared nucleotide substitutions, and all other species by their unique combination: G (79, except some *Anelosimus lamarcki* sp. n.), G (184, except *Anelosimus buffoni* sp. n. and *Anelosimus wallacei* sp. n.), G (202, except most *Anelosimus ata* sp. n.), T (479, except *Anelosimus nazariani*), G (511, except *Anelosimus buffoni* sp. n.and *Anelosimus wallacei* sp. n.), T (553, except *Anelosimus tita*), T (709, except *Anelosimus ata*), G (772, except *Anelosimus lamarcki*), (796, except *Anelosimus buffoni* and some *Anelosimus may*), G (838, except *Anelosimus darwini*).

###### Distribution.

Only known from type locality.

###### Natural history.

As in other species of this group a female can be found in its web with close to 50 juveniles and juveniles appear to cohabit in the web until close to adulthood.

##### Anelosimus torfi

Taxon classificationAnimaliaAraneaeTheridiidae

Agnarsson sp. n.

http://zoobank.org/77C3E795-7A2A-4163-87E9-408C92BB5D19

Fig. 7

###### Type material.

**Holotype** female from Ambohitantely Special Reserve (18.161°S, 47.302°E), 1500 m alt, Analamanga region, Ankazobe district, Madagascar, 28.iv.2008, montane forest, col. Agnarsson and Kuntner, in NMNH.

###### Other material.

Only known from holotype.

###### Etymology.

The species epithet is a noun in apposition and honors Torfi Agnarsson, the senior author´s brother.

###### Diagnosis.

*Anelosimus torfi* can be diagnosed from all other *Anelosimus* based on the distinctly dark coloration and from all but *Anelosimus vondrona* based on its pendulum-like septum. *Anelosimus torfi* can be diagnosed from other Madagascan *Anelosimus* on the basis of the following unique mtDNA nucleotide substitutions at the following standard DNA barcode alignment positions: G (43), C (620), A (764), G (952), T (953), G (955). It can also be readily diagnosed from most other *Anelosimus* based the following partially shared nucleotide substitutions, and all other species by their unique combination: A (46, except *Anelosimus nazariani*), A (256, except *Anelosimus salut* and *Anelosimus hookeri*), T (364, except *Anelosimus darwini*), T (370, except *Anelosimus salut*), T (412, except *Anelosimus salut*), A (469, except *Anelosimus salut*), T (484, except *Anelosimus may* and *Anelosimus nazariani*), A (622, except *Anelosimus salut*), G (625, except *Anelosimus ata* and *Anelosimus huxleyi*), A (754, except *Anelosimus salut*), G (817, except *Anelosimus huxleyi*), G (841, except *Anelosimus nazariani*), T (940, except *Anelosimus salut*), A (943, except *Anelosimus moramora*), A (961, except *Anelosimus salut*).

**Figure 7.**
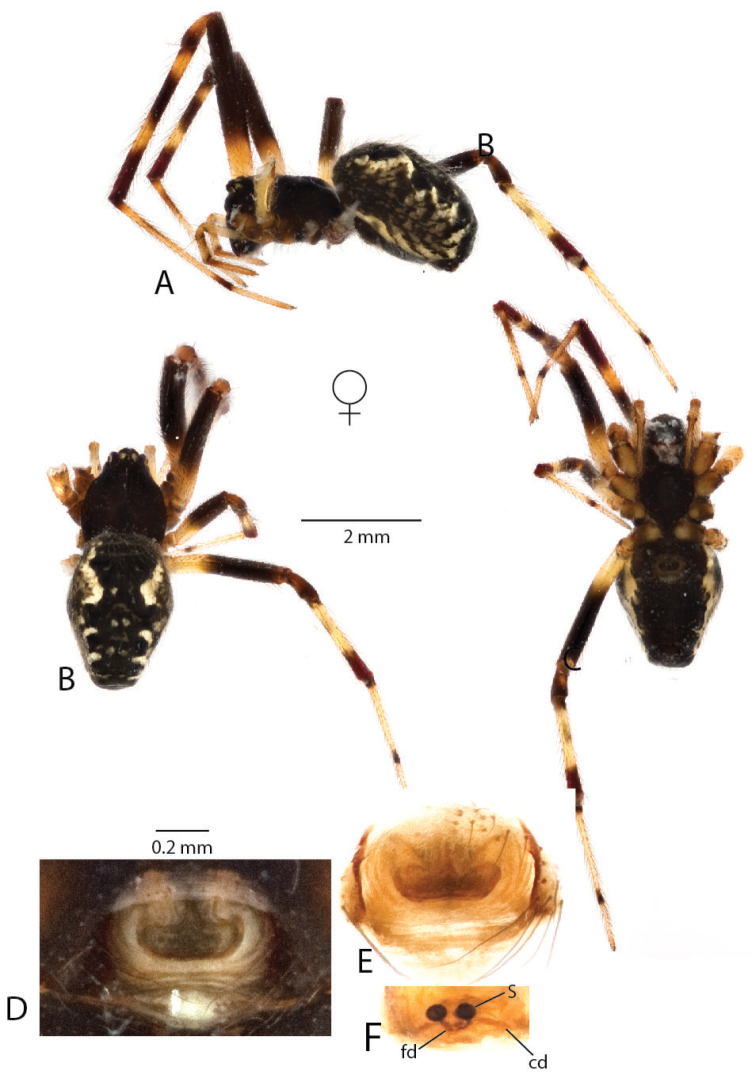
*Anelosimus torfi*: **A–C** female lateral, dorsal, ventral **D** epigynum ventral view **E–F** cleared epigynum, ventral, dorsal.

###### Description.

*Female*: Total length 4.1. Cephalothorax 1.95 long, 1.4 wide, 1.06 high, dark black-brown. Sternum 1.13 long,. 99 wide, extending half way between coxae IV, brown. Abdomen 2.67 long, 1.76 wide, 1.67 high.black base with yellow patterns. Eyes subequal in size about 0.12 in diameter. Clypeus height about 2.1 times one AME diameter. Chelicerae with one large tooth, three denticles not visible on specimen. Leg I femur 2.21, patella 0.76, tibia 2.81, metatarsus 2.47, tarsus 1.08. Leg formula 3214, with leg 4 significantly longer than leg 1. Legs primarily black-brown with yellow bands, dark at junction between each leg segment. 4 small trichobothria dorsally on all tibia. Trichobothria on all metatarsi (2), single tricobothria on tarsi. Four dorsal trichobothria on female palpal tibia.

*Variation*: Total length 4.1–4.32. Abdomen 2.67–2.84 long, 1.76–1.9 wide, 1.67–2.04 high. Femur 2.21–2.47.

###### Distribution.

Only known from type locality.

###### Natural history.

Unknown, predicted to be subsocial.

##### Anelosimus hookeri

Taxon classificationAnimaliaAraneaeTheridiidae

Agnarsson, Kuntner & Jencik sp. n.

http://zoobank.org/1A2C51AA-F24E-4E60-8167-E948A507F3AF

Fig. 8

###### Type material.

**Holotype** female from Ambohitantely Special Reserve (18.161°S, 47.302°E), 1500 m alt, Analamanga region, Ankazobe district, Madagascar, 28.iv.2008, montane forest, col. Agnarsson and Kuntner, in NMNH.

###### Other material.

Only known from holotype.

###### Etymology.

The species epithet is a noun in genitive case that honors the evolutionary biologist Joseph Dalton Hooker, who was among the first scientists to publish work announcing support for Darwin´s theory of evolution by natural selection.

###### Diagnosis.

**Figure 8.**
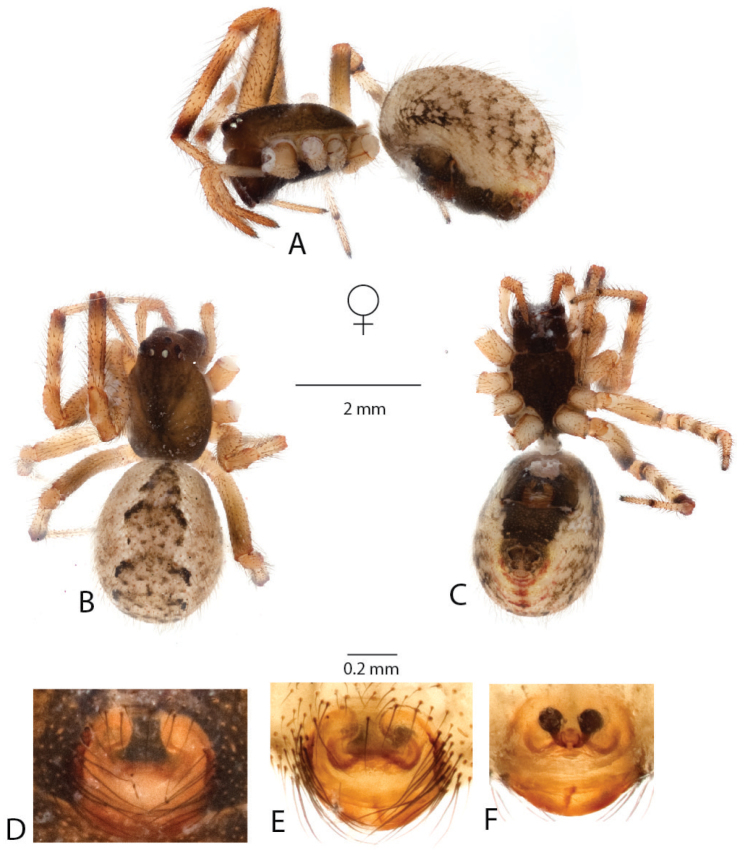
*Anelosimus hookeri*: **A–C** female lateral, dorsal and ventral views **D** epigynum ventral view **E–F** cleared epigynum ventral, dorsal.

*Anelosimus hookeri* differs from all other *Anelosimus* by the combination of pale coloring (Fig. 8A–C), and a pendulum-like septum that is widest at its extremes (Fig. 8E). *Anelosimus hookeri* can be diagnosed from other Madagascan *Anelosimus* on the basis of the following unique mtDNA nucleotide substitutions at the following standard DNA barcode alignment positions: G (85), G (479). It can also be readily diagnosed from most other *Anelosimus* based the following partially shared nucleotide substitutions, and all other species by their unique combination: T (22, except *Anelosimus tita* and *Anelosimus huxleyi*), G (100, except *Anelosimus may*, and some *Anelosimus darwini*), A (256, except *Anelosimus torfi* and *Anelosimus salut*), G (379, except *Anelosimus wallacei*), G (466, except *Anelosimus vondrona*), G (487, except *Anelosimus ata*), G (514, except *Anelosimus lamarcki* and most *Anelosimus vondrona*).

###### Description.

*Female*: Total length 4.76. Cephalothorax 2.17 long, 1.43 wide, 1.26 high, dark brown. Sternum 1.26 long, 0.99 wide, extending half way between coxae IV, brown. Abdomen 2.99 long, 2.05 wide, 2.17 high. White base with black/brown spots, red marks near spinnerets, dark brown around genitalia. Eyes subequal in size about 0.12 in diameter. Clypeus height about 2.1 times one AME diameter. Chelicerae with one large tooth, three denticles prolaterally. Leg I femur 1.76, patella 0.63, tibia 1.99, metatarsus 1.89, tarsus 0.88. Leg formula 1243, with leg 2 slightly longer than leg 1 and leg 3 slightly longer than leg 4. Leg light orange-brown, with alternating light and dark shaded bands, and very dark at metatarsus/tarsus junction and distal tip of tarsus. Numerous (4–5) small trichobothria dorsally on all tibia, 4 on tibia II, 5 on tibia I. Trichobothria on all metatarsi (2–3). Four dorsal trichobothria on female palpal tibia.

*Variation*: only known from holotype.

###### Distribution.

Only known from type locality.

###### Natural history.

Unknown, predicted to be subsocial.

##### Anelosimus lamarcki

Taxon classificationAnimaliaAraneaeTheridiidae

Agnarsson & Goh sp. n.

http://zoobank.org/1A46E0A6-99BE-4AFE-BDE1-5FD9C96E36C1

Fig. 9

###### Type material.

**Holotype** female from Ranamofana National Park (21.25°S, 47.43°E), montane rainforest, 9801050 m alt, 27.iv.–2.v.2013, col. Pruitt, in NMNH.

###### Other material.

Same locality and collection, several adult females.

###### Etymology.

The species epithet is a noun in genitive case that honors the early evolutionary biologist Jean-Babtiste Lamarck, the first scientists to develop a thorough and coherent evolutionary theory, though it was later shown by Darwin to be flawed in major ways.

###### Diagnosis.

*Anelosimus lamarcki* can be diagnosed from other Madagascan *Anelosimus* by the heavily sclerotized copulatory ducts and small spermathecae that barely exceed the diameter of the copulatory ducts. *Anelosimus lamarcki* can be diagnosed from other Madagascan *Anelosimus* on the basis of the following unique mtDNA nucleotide substitutions at the following standard DNA barcode alignment positions: G (280), C (562). It can also be readily diagnosed from most other *Anelosimus* based the following partially shared nucleotide substitutions, and all other species by their unique combination: G (502, except rarely in *Anelosimus may*), G (514, except *Anelosimus hookeri* and most *Anelosimus vondrona*), G (553, except some *Anelosimus huxleyi*), G (766, except some *Anelosimus may*), G (772, except *Anelosimus andasibe*), G (814, except most *Anelosimus vondrona*).

**Figure 9.**
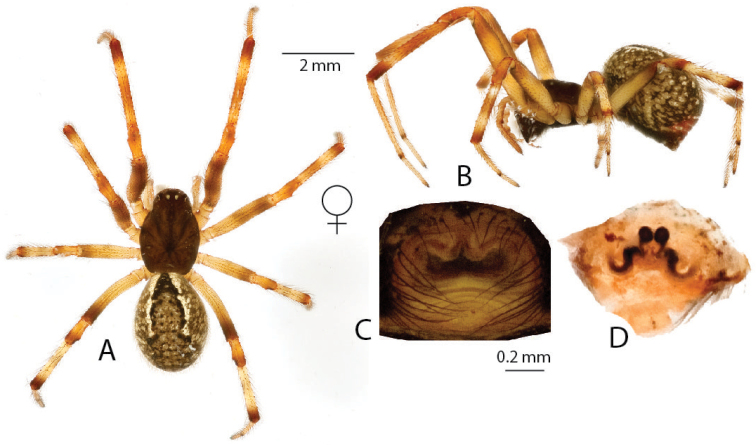
*Anelosimus lamarcki*: **A–B** female dorsal and lateral views **C** epigynum ventral view **D** epigynum cleared dorsal.

###### Description.

*Female* (holotype): Total length 5.16. Cephalothorax 2.32 long, 1.70 wide, 0.98 high, dark brown. Abdomen 2.88 long, 2.04 wide, 1.90 high. Light brown base with black/white spots, black and white longitudinal band extending just beyond half of abdomen, red marks near spinnerets. Eyes subequal in size about 0.14 in diameter. Leg I femur 2.77, patella 0.84, tibia 2.34, metatarsus 2.28, tarsus 0.91. Leg formula 1423. Leg light orange-brown, with alternating light and dark shaded bands, and very dark at distal tips of femur, patella, tibia and metatarsus. Numerous (6 – 7) small trichobothria dorsally on all tibia, 7 on tibia I, 6 on tibia II, 7 on tibia III, 6 on tibia IV.

*Variation*: Total length 5.00–6.80. Prosoma 2.30–2.90 long. Abdomen 2.70–3.20 long. Femur I 2.70–3.20.

###### Distribution.

Only known from type locality.

###### Natural history.

We sampled twelve colonies of *Anelosimus lamarcki*. Colonies were found both along trails in the forest interior and along roadsides and ornamental shrubbery. The ten colonies in the forest interior contained females with groups of small juveniles, likely instars I–II, and colonies along road sides contained one penultimate or mature female. Like *Anelosimus vondrona*, *Anelosimus lamarcki* webs contained an impressive diversity of foreign spiders including multiple theridiids, saliticids, sparassids, a thomisid, and several linyphiids. We observed multiple co-feedings events between *Anelosimus lamarcki* and its web associates during staged prey capture events. Whether *Anelosimus lamarcki* or its web associate was the first to subdue the prey differed across trials.

##### Anelosimus tita

Taxon classificationAnimaliaAraneaeTheridiidae

Agnarsson, Kuntner & Jencik sp. n.

http://zoobank.org/85C991FF-0021-4D6E-80A0-65813CCD3F5E

Fig. 10

###### Type material.

**Holotype** female from Ambohitantely Special Reserve (18.161°S, 47.302°E), 1500 m alt, Analamanga region, Ankazobe district, Madagascar, 28.iv.2008, montane forest, col. Agnarsson and Kuntner, in NMNH.

###### Other material.

Only known from holotype.

###### Etymology.

The species is a noun in apposition named in the honor of the first author’s mother-in-law Yadira Collado Ulloa, affectionately known to her grandchildren as ‘Tita’.

###### Diagnosis.

**Figure 10.**
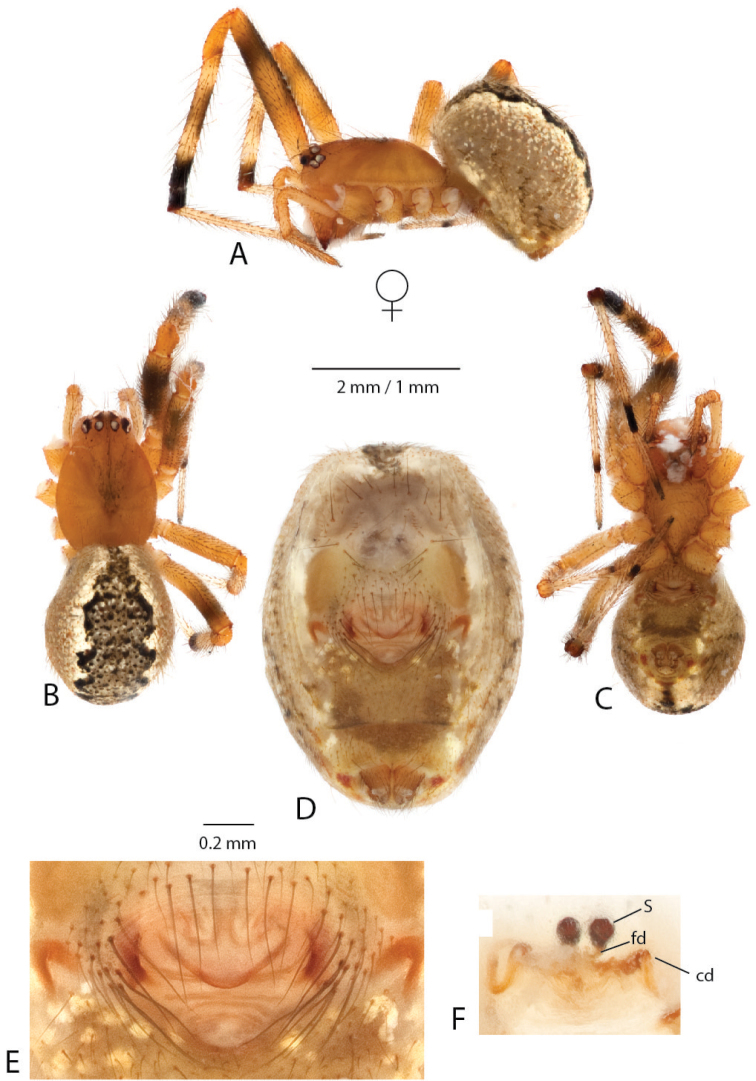
*Anelosimus tita*: **A–D** female lateral, dorsal, ventral, and ventral abdomen **E** epigynum ventral **F** epigynum cleared, dorsal.

*Anelosimus tita* can be diagnosed from other Madagascan *Anelosimus* by the triangular shape of the septum (Fig. 10E) and on the basis of the following unique mtDNA nucleotide substitutions at the following standard DNA barcode alignment positions: T (30), G (37), T (80), T (81), T(82), A(83), G (109), G (214), T(220), G (319), T (328), T (586), T (625), G (873), G (883), A (903), G (919). It can also be readily diagnosed from most other *Anelosimus* based the following partially shared nucleotide substitutions, and all other species by their unique combination: T (22, except *Anelosimus hookeri* and *Anelosimus huxleyi*), T (121, except *Anelosimus nazariani*), G (190, except some *Anelosimus huxleyi*), G (262, except *Anelosimus nazariani*), T (532, except some *Anelosimus huxleyi*), T (553, except *Anelosimus andasibe*).

###### Description.

*Female*: Total length 3.87 Cephalothorax 1.9 long, 1.34 wide, 1.09 high, brown. Sternum 1.02 long, 0.87 wide, extending half way between coxae IV, orange. Abdomen 2.44 long, 1.68 wide, 1.33 high. Mixed pattern of white, grey, and black. Eyes subequal in size about 0.11 in diameter. Clypeus height about 2 times one AME diameter. Chelicerae with one large tooth, three denticles prolaterally. Leg I femur 2.01, patella 0.66, tibia 2.02, metatarsus 1.84, tarsus 0.91. Leg formula 3421, with leg 1 significantly longer than leg 2. Legs alternating between light orange and dark brown bands. 3–4 small trichobothria dorsally on tibia, 3 on tibia 1. 3 or 4 dorsal trichobothria on female palpal tibia.

*Variation*: only known from holotype.

###### Distribution.

Only known from type locality.

###### Natural history.

Unknown, predicted to be subsocial.

##### Anelosimus ata

Taxon classificationAnimaliaAraneaeTheridiidae

Agnarsson, Kuntner & Jencik sp. n.

http://zoobank.org/E5EE186A-D324-413B-AD04-8F395EFB6EA6

Fig. 11

###### Notes.

In 2005 we described *Anelosimus may* Agnarsson, based on a holotype male and females both from Ambohitantely and Périnet (Agnarsson and Kuntner 2005). Here we establish based on DNA analyses that *Anelosimus may* as currently circumscribed contains two species. *Anelosimus may* is indeed as originally thought found both in Ambohitantely and Périnet, while the very similar species described here, *Anelosimus ata* is so far restricted to Périnet.

###### Type material.

**Holotype** female from Périnet Special Reserve (P.N. Andasibe Mantadia), Toamasina Province, Madagascar, (18.935°S, 48.418°E), 7–8.v.2001, montane forest, 900–1000 m, (I. Agnarsson and M. Kuntner) (NMNH), based on the paratype originally attributed to *Anelosimus may*, see Agnarsson and Kuntner (2005) p. 580.

###### Other material.

Several female specimens from same locality.

###### Etymology.

The species epithet is a noun in apposition named in the honor of the first author’s father-in-law Jorge May-Barquero, affectionately known to his grandchildren as ‘Ata’.

###### Diagnosis.

**Figure 11.**
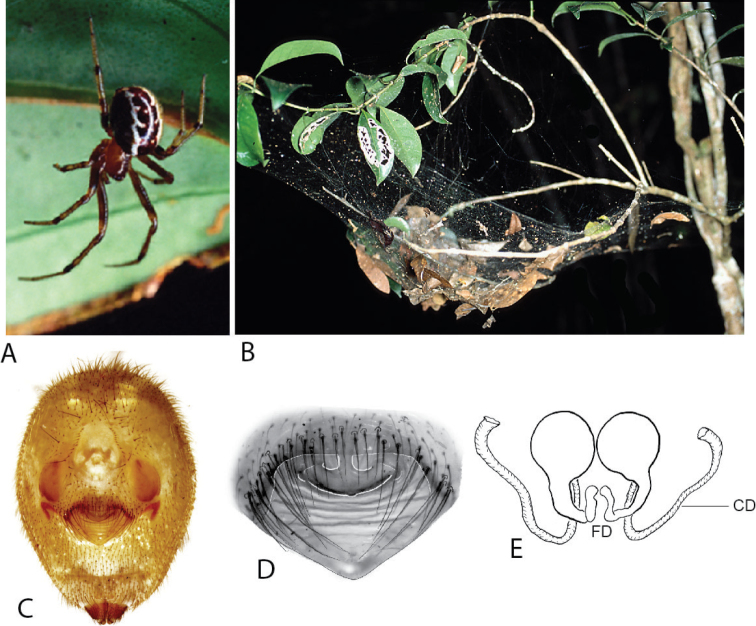
*Anelosimus ata*: **A–B** female and her web **C** female abdominal venter **D** epigynum, ventral **E** epigynum, dorsal. Images are schematic and not to scale.

*Anelosimus ata* can be diagnosed from all other *Anelosimus*, expect *Anelosimus may*, by the anchor-shaped septum (Fig. 11D) and from A. may by the juxtaposed spermathecae and the pathway of the copulatory ducts following the septum edge (Fig. 11E). *Anelosimus ata* can be diagnosed from other Madagascan *Anelosimus* on the basis of the following unique mtDNA nucleotide substitutions at the following standard DNA barcode alignment positions: A (88), G (166), A(169), G (253), G (358), T (835), G (910). It can also be readily diagnosed from most other *Anelosimus* based the following partially shared nucleotide substitutions, and all other species by their unique combination: T (127, except *Anelosimus nazariani* and *Anelosimus darwini*), T (181, except *Anelosimus may*), T (355, except *Anelosimus may*), G (487, except *Anelosimus hookeri*), G (625, except *Anelosimus torfi* and *Anelosimus huxleyi*), T (709, except *Anelosimus andasibe*), G (751, except some *Anelosimus may*).

###### Description.

*Female*: Total length 5.01. Cephalothorax 2.28 long, 1.82 wide, 1.45 high, brown. Sternum 1.35 long, 1.16 wide, extending halfway between coxae IV, brown. Abdomen 2.93 long, 2.44 wide, 2.52 high. Pattern as in Fig. 3A. Eyes subequal in size about 0.12 in diameter. Clypeus height about 2.4 one AME diameter. Chelicerae with one large and two small prolateral teeth, three denticles retrolaterally. Leg I femur 2.89, patella 0.98, tibia 2.70, metatarsus 2.57, tarsus 0.94. Femur about 5 longer than wide, metatarsus I about 16longer than wide. Leg formula 1243, with leg II very slightly longer than leg IV. Leg base colour as carapace, light orange-brown, with distal tip of tibia darkened, and metatarsus/tarsus junction dark. Tarsal organs slightly distal (0.55–0.60) on tarsi I and II, central (0.5) on III, slightly proximal (0.45) on IV, distal (0.85) on female palp, positions vary slightly between specimens. Numerous (seven to eight) small trichobothria dorsally on all tibia, seven on tibia III, eight on tibia I. Trichobothria on metatarsi I–III central or slightly proximal (about 0.45–0.50), absent on metatarsus IV. Four to five dorsal trichobothria on female palpal tibia.

*Variation*: female total length 4.90–5.15.

###### Distribution.

Only known from type locality.

###### Natural history.

This species is common at its type locality and webs have been found with females and up to 80 spiderlings. Juveniles cohabit in the web with the mother until she dies and appear to disperse close to adulthood. Webs without adult females generally contained instar 4–6 juveniles.

##### Anelosimus darwini

Taxon classificationAnimaliaAraneaeTheridiidae

Agnarsson, Kuntner & Jencik sp. n.

http://zoobank.org/DAA2F5AE-0AF1-49A4-82B5-72F79AB99333

Fig. 12

###### Type material.

**Holotype** female holotype from Ambohitantely Special Reserve (18.197°S, 47.285°E), 1600 m alt, Analamanga region, Ankazobe district, Madagascar, 28.iv.2008, montane forest, col. Agnarsson and Kuntner, in NMNH.

###### Other material.

Juveniles from same locality.

###### Etymology.

The species epithet is a noun in the genitive case and honors Charles Darwin, the father of evolutionary biology.

###### Diagnosis.

**Figure 12.**
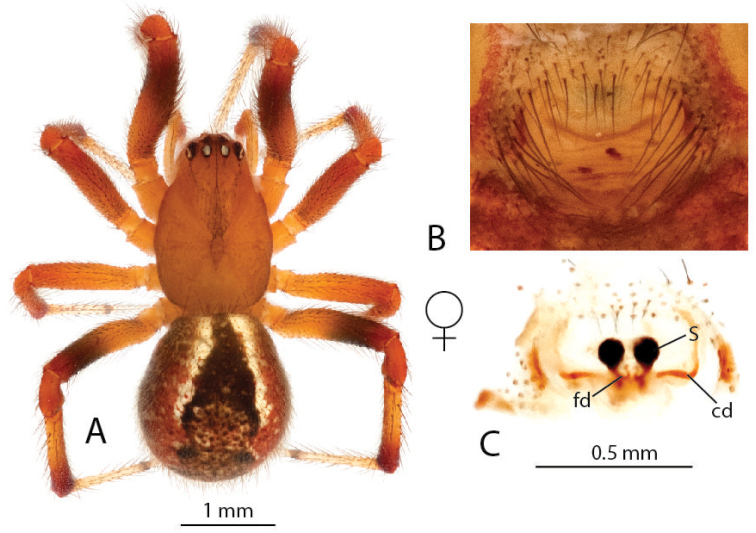
*Anelosimus darwini*: **A** female dorsal **B** epigynum, ventral **C** epigynum cleared, dorsal.

*Anelosimus darwini* can be diagnosed from all other *Anelosimus*, expect *Anelosimus may*, and *Anelosimus ata*, by the anchor-shaped septum (Fig. 12B) and from *Anelosimus may* and *Anelosimus ata* by the pathway of the copulatory duct with a near 90° bend (Fig. 12C). *Anelosimus darwini* can be diagnosed from other Madagascan *Anelosimus* on the basis of the following unique mtDNA nucleotide substitutions at the following standard DNA barcode alignment positions: T (84), T (190), T (526), A (848). It can also be readily diagnosed from most other *Anelosimus* based the following partially shared nucleotide substitutions, and all other species by their unique combination: T (127, except *Anelosimus nazariani* and *Anelosimus ata*), T (130, except *Anelosimus nazariani*), A (133, except *Anelosimus huxleyi*), G (229, except some *Anelosimus may*), G (244, except *Anelosimus may*), T (352, except *Anelosimus may* and *Anelosimus sallee*), T (364, except *Anelosimus torfi*), G (556, except *Anelosimus nazariani*), T (631, except *Anelosimus salut*), G (838, except *Anelosimus andasibe*).

###### Description.

*Female* (holotype): Total length 3.70. Cephalothorax 1.88 long, 1.27 wide, 0.93 high. Red-brown. Sternum 1.04 long, 0.82 wide, extending between coxae IV, dark brown. Abdomen 1.79 long, 1.63 wide, 2.45 high. Brown pattern with 2 white streaks. Eyes subequal in size about 0.11 in diameter. Chelicerae each with 1 large tooth, 3 denticles located prolaterally. Clypeus height about 2.3 times one AME diameter. Leg I femur 2.10, patella 0.62, tibia 1.86, metatarsus 1.49, tarsus 0.93. Legs roughly same color as cephalothorax. Leg formula 1432. Numerous (3–4) small trichobothria dorsally on all tibiae. 2–3 trichobothria on metatarsus, absent on tarsus.

*Variation*: only known from holotype.

###### Distribution.

Only known from type locality.

###### Natural history.

Unknown, predicted to be subsocial.

##### Anelosimus wallacei

Taxon classificationAnimaliaAraneaeTheridiidae

Agnarsson, Veve & Kuntner sp. n.

http://zoobank.org/46C8F33C-EE8C-4844-9F07-4A66D0F9110C

Fig. 13A–E

###### Type material.

**Holotype** female from Périnet Special Reserve (P.N. Andasibe Mantadia), Toamasina Province, Madagascar, (18.935°S, 48.418°E), 12–28.xi.2008, montane forest, 900–1000 m, col Hanitriniaina, in NMNH.

###### Other material.

Additional specimens from same locality, 3–20.iv.2008 and 12–28.xi.2008, col. Agnarsson, Kuntner, and Hanitriniaina.

###### Etymology.

The species epithet is a noun in the genitive case and honors the evolutionary biologist Alfred Russel Wallace, a contemporary of Darwin and co-author of the first paper on natural selection.

###### Diagnosis.

**Figure 13.**
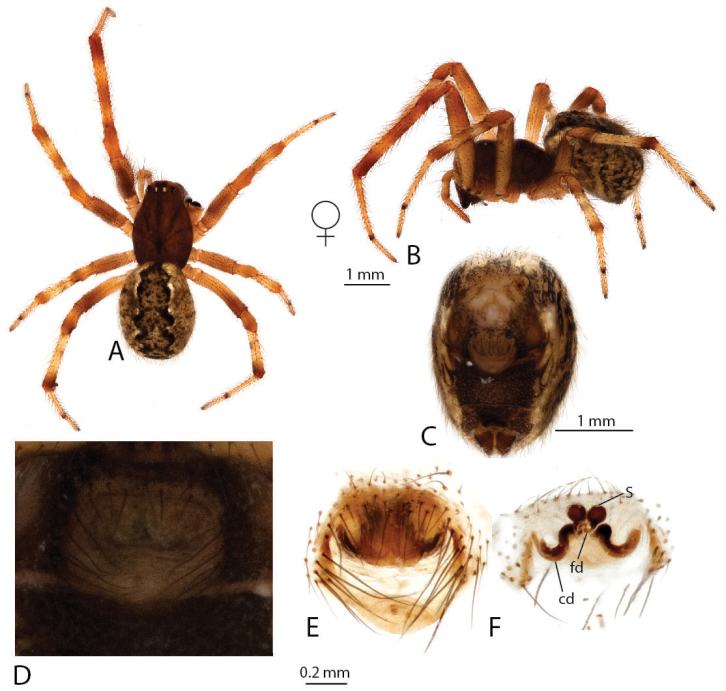
*Anelosimus wallacei*: **A–B** female dorsal and lateral views **C** abdomen ventral **D** epigynum ventral view **E** epigynum cleared ventral **F** epigynum cleared, dorsal.

*Anelosimus wallacei* can be diagnosed from all other *Anelosimus*, expect *Anelosimus andasibe* and *Anelosimus buffoni* by the W pattern of the septum (Fig. 13C) and from *Anelosimus andasibe* and *Anelosimus buffoni* by substitutions A (241), G (379) and G (745). *Anelosimus wallacei* can be diagnosed from other Madagascan *Anelosimus* on the basis of the following unique mtDNA nucleotide substitutions at the following standard DNA barcode alignment positions: C (283), G (679). It can also be readily diagnosed from most other *Anelosimus* based the following partially shared nucleotide substitutions, and all other species by their unique combination: G (184, except *Anelosimus buffoni* and *Anelosimus andasibe*), G (379, except *Anelosimus hookeri*), G (511, except *Anelosimus buffoni* and *Anelosimus andasibe*), G (745, expect *Anelosimus nazariani* and some *Anelosimus ata*).

###### Description.

*Female*: Total length 4.72 Cephalothorax 2.14 long, 1.57 wide, 0.44 high. Sternum 1.26 long, 1.05 wide, extending halfway between coxae IV, dark brown. Abdomen 2.58 long, 2.01 wide, 1.78 high, color and pattern as in Fig. 13A. Eyes subequal in size about 0.13 in diameter. Clypeus height about 2 times one AME diameter. Chelicerae with one large tooth, three denticles retrolaterally. Leg 1 femur 2.35, patella 0.88, tibia 3.13, metatarsus 1.49, tarsus 0.76. Leg formula 1243. Legs light brown-yellow with brown at junctions between tibia and metatarsus, and metatarsus and tarsus. 5 small trichobrothia dorsally on tibiae, two dorsally on metatarsi.

*Variation*: Total length 4.72–4.8. Cephalothorax 2.14–2.25 long. Femur I 1.76–2.35.

###### Distribution.

Only known from type locality.

###### Natural history.

This species occurs almost exclusively in closed forest at its type locality. Like other species of the group it makes webs with females and spiderlings cohabiting, with up to 83 spiderlings found in a single web. Webs without adult females generally contained instar 4–6 juveniles.

##### Anelosimus huxleyi

Taxon classificationAnimaliaAraneaeTheridiidae

Agnarsson, Veve & Kuntner sp. n.

http://zoobank.org/CB0DEF79-3C74-4755-AB91-4A7EAD86CEE6

Fig. 14

###### Type material.

**Holotype** female from Périnet Special Reserve (P.N. Andasibe Mantadia), Toamasina Province, Madagascar, (18.935°S, 48.418°E), 12–28.xi.2008, montane forest, 900–1000 m, col Hanitriniaina, in NMNH.

###### Other material.

Additional specimens from same locality, 3–20.iv.2008 and 12–28.xi.2008, col. Agnarsson, Kuntner, and Hanitriniaina.

###### Etymology.

The species epithet is a noun in the genitive case and honors the evolutionary biologist Thomas Henry Huxley; ‘Darwin´s bulldog’.

###### Diagnosis.

**Figure 14.**
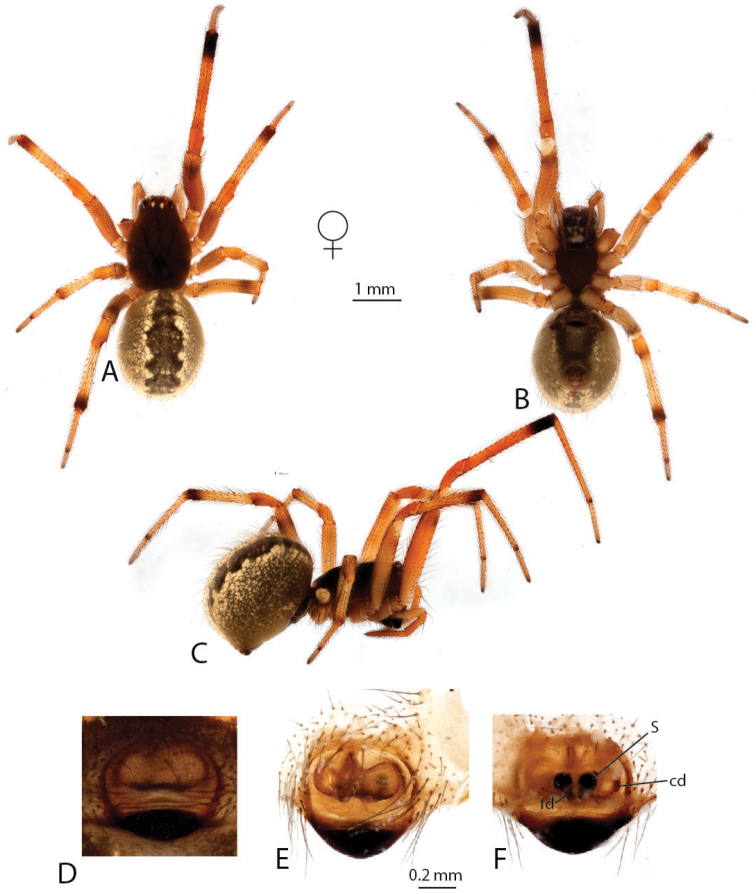
*Anelosimus huxleyi*: **A–C** female dorsal, ventral and lateral views **D** epigynum ventral view **E–F** cleared epigynum ventral, dorsal.

*Anelosimus huxleyi* females can be diagnosed from all other species except *Anelosimus vondrona*, by the relatively broad septum that extends the entire width of the epigynum (Fig. 14D–E) and from *Anelosimus vondrona* by the more heavily sclerotized lower margin of the epigynal plate (Fig. 14D–F). *Anelosimus huxleyi* can be diagnosed from other Madagascan *Anelosimus* on the basis of the following unique mtDNA nucleotide substitutions at the following standard DNA barcode alignment positions: A (283), A (418), T (760), G (784). It can also be readily diagnosed from most other *Anelosimus* based the following partially shared nucleotide substitutions, and all other species by their unique combination: T (22, except *Anelosimus tita* and *Anelosimus hookeri*), T (58, except *Anelosimus may*), A (133, except *Anelosimus darwini*), G (181), T (139, except *Anelosimus sallee*), G (619, except *Anelosimus vondrona*), G (625, except *Anelosimus torfi* and *Anelosimus ata*), T (781, except *Anelosimus may* and *Anelosimus salut*), G (817, except *Anelosimus torfi*), T (838, except *Anelosimus sallee*).

###### Description.

*Female*: Total length 5.64 Cephalothorax 2.54 long, 1.79 wide, 0.63 high. Sternum 1.30 long, 1.11 wide, extending halfway between coxae IV, dark brown. Abdomen 3.10 long, 2.45 wide, 2.63 high, color and pattern as in Fig. 14A. Eyes subequal in size about 0.16 in diameter. Clypeus height about 2 times one AME diameter. Chelicerae with one large tooth, 3-4 denticles retrolaterally. Leg 1 femur 3.17, patella 0.85, tibia 2.75, metatarsus 2.38, tarsus 1.07. Leg formula 1243. Legs are light brown-yellow with dark brown at junctions between tibia and metatarsus, and metatarsus and tarsus. 5 small trichobothria dorsally on tibiae, 4 dorsally on metatarsi.

*Variation*: Total length 5.50–5.70. Cephalothorax 2.50–2.55 long. Femur I 3.10–3.20.

###### Distribution.

Only known from type locality.

###### Natural history.

As in other species of this group a female can be found in its web with close to 50 juveniles and juveniles appear to cohabit in the web until close to adulthood.

##### Anelosimus buffoni

Taxon classificationAnimaliaAraneaeTheridiidae

Agnarsson, Kuntner & Jencik sp. n.

http://zoobank.org/F0FFD4BF-E1F6-446B-9996-184EA7D846B7

Fig. 15

###### Notes.

Unfortunately most adult specimens of this species, almost all of which were collected in April 2008, were lost during the transport of the Agnarsson lab from UPR to UVM. Of the only two remaining adult females the carapace and legs had been consumed for DNA extraction, though total length was measured before specimens were processed for DNA extraction. The species description is therefore abbreviated and limited to the abdomen, genitalia, and DNA barcode.

###### Type material.

**Holotype** and paratype female from Périnet Special Reserve (P.N. Andasibe Mantadia), Toamasina Province, Madagascar, (18.935°S, 48.418°E), 12–28.xi.2008, montane forest, 900–1000 m, col Hanitriniaina, in NMNH.

###### Etymology.

The species epithet is a noun in the genitive case and honors the great naturalist Georges-Louis Leclerc, Comte de Buffon.

###### Diagnosis.

**Figure 15.**
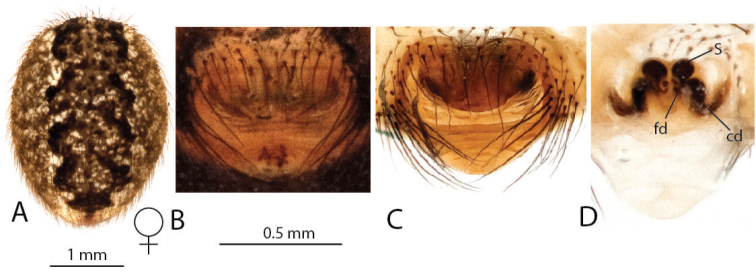
*Anelosimus buffoni*: **A** female abdomen dorsal **B** epigynum ventral view **C** epigynum cleared ventral **D** epigynum cleared dorsal.

*Anelosimus buffoni* females can be diagnosed from all other species expect *Anelosimus andasibe* and *Anelosimus wallacei* by the W pattern of the septum (Fig. 15B–C) and from *Anelosimus wallacei* by lacking substitution A (241), from *Anelosimus andasibe* by having substitution G (349), and by both by having substitution A (559). *Anelosimus buffoni* can be diagnosed from other Madagascan *Anelosimus* on the basis of the following unique mtDNA nucleotide substitutions at the following standard DNA barcode alignment positions: G (742), T (769). It can also be readily diagnosed from most other *Anelosimus* based the following partially shared nucleotide substitutions, and all other species by their unique combination: G (364, except some *Anelosimus salut*), G (184, except *Anelosimus wallacei* and *Anelosimus andasibe*), G (511, except *Anelosimus andasibe* and *Anelosimus wallacei*), G (796, except *Anelosimus andasibe* and some *Anelosimus may*), G (799, except *Anelosimus vondrona*).

###### Description.

Total length 4.1, Abdomen 2.59 long, 1.96 wide, color and pattern as in Fig. 15A.

###### Distribution.

Only known from type locality.

###### Natural history.

As in other species of this group a female can be found in its web with close to 50 juveniles and juveniles appear to cohabit in the web until close to adulthood.

##### Anelosimus moramora

Taxon classificationAnimaliaAraneaeTheridiidae

Agnarsson, Kuntner & Jencik sp. n.

http://zoobank.org/4404333D-C40F-458E-AC01-7FFF94EC9D4D

Fig. 16

###### Type material.

**Holotype** female from Montagne d’Ambre National Park (12.516972°S, 49.178778°E), 1005 m alt, Antsiranana district, Madagascar, 4.iv.2008, montane forest, col. Agnarsson and Kuntner, in NMNH.

###### Etymology.

The species epithet is a Madagascan aphorism or motto meaning ‘no rush’ or ‘take it easy’.

###### Diagnosis.

*Anelosimus moramora* females can be diagnosed from all other species except *Anelosimus ata* by the relatively small and pointy shape of the septum and from *Anelosimus ata* by its smaller size. *Anelosimus moramora* can be diagnosed from other Madagascan *Anelosimus* on the basis of the following unique mtDNA nucleotide substitutions at the following standard DNA barcode alignment positions (note that only a short fragment of the divergent *Anelosimus moramora* barcode is available starting at position 824): C (843), C (888), T (897), A (901), C (906), A (914), C (924), C (939), C (967), A (979). It can also be diagnosed from all *Anelosimus* except *Anelosimus torfi* based on A (943).

**Figure 16.**
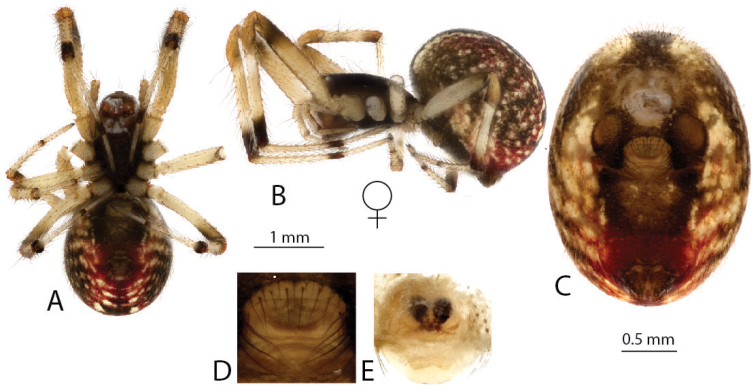
*Anelosimus moramora*: **A–C** female ventral, lateral and abdomen ventral **D** epigynum ventral view **E** epigynum cleared, dorsal.

###### Description.

*Female*: Total length 3.31. Cephalothorax 1.4 long, 1.06 wide, 0.87 high, brown. Sternum 0.78 long, 0.67 wide, extending half way between coxae IV, brown. Abdomen 2.02 long, 1.6 wide, 2.26 high. Red-brown with 2 white streaks. Eyes subequal in size about 0.09 in diameter. Clypeus height about 2.5 times one AME diameter. Chelicerae with one large tooth, three denticles prolaterally. Leg I femur 1.67, patella 0.5, tibia 1.41, metatarsus 1.1, tarsus 0.65. Leg formula 1243. Legs light brown-yellow with dark brown at junctions between tibia and metatarsus, and metatarsus and tarus. 4 small trichobothria dorsally on tibiae, 3 on all metatarsi. 3–4 dorsal trichobothria on female palpal tibia.

*Variation*: only known from holotype.

###### Distribution.

Only known from type locality.

###### Natural history.

Unknown, predicted to be subsocial.

##### Anelosimus decaryi

Taxon classificationAnimaliaAraneaeTheridiidae

(Fage, 1930)

Fig. 17

###### Notes.

*Anelosimus decaryi* does not belong to the ‘Madagascar group´ of subsocial montane species but rather to the ´solitary clade´ of *Anelosimus* (see Agnarsson et al. 2010). It was recently redescribed (see Agnarsson et al. 2010) and is here only re-illustrated for completeness so that all *Anelosimus* species currently known to occur in Madagascar can be identified from a single source.

**Figure 17.**
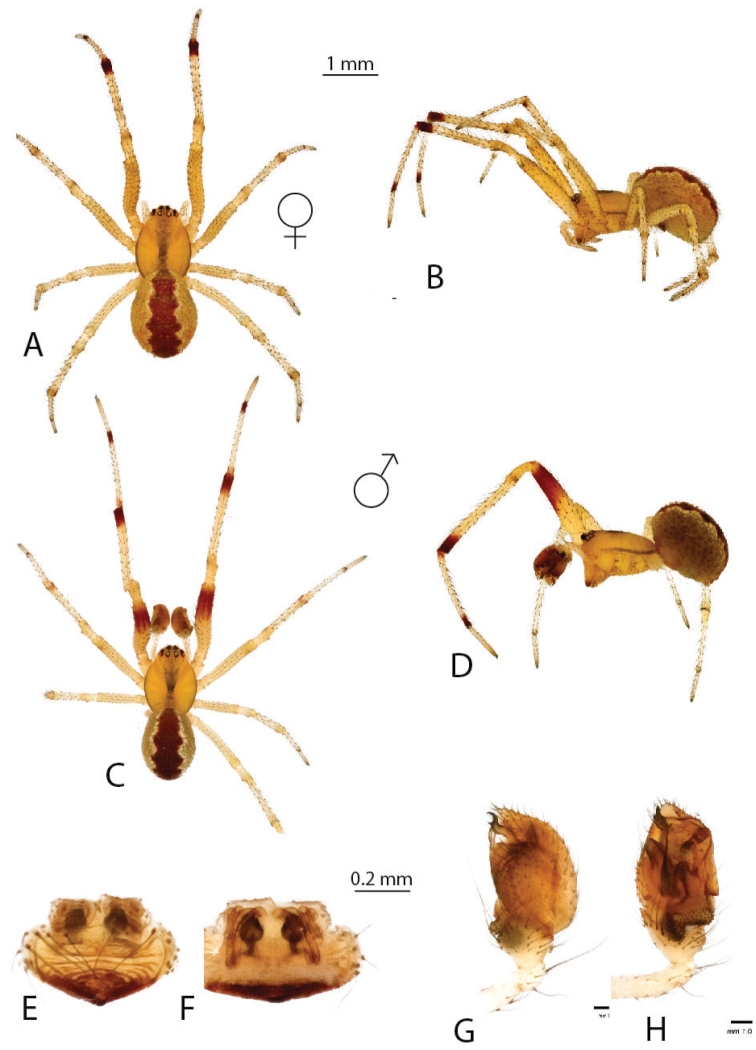
*Anelosimus decaryi*: **A–B** female dorsal, lateral **C–D** male dorsal, lateral **E–F** epigynum ventral, dorsal **G–H** male palp mesal, ventral.

###### Distribution.

Orangea Peninsula (N Madagascar), Ranamofana (Central Madagascar), Comoros Islands, and Aldabra atoll.

###### Natural history.

*Anelosimus decaryi* is solitary with very brief cohabitation between mother and offspring, which disperse at early instars (Agnarsson et al. 2010). The species was previously thought to be restricted to coastal habitats in the north, however, it also occurs in Ranamofana. Seven colonies of *Anelosimus decaryi* were sampled along the riparian zones directly flanking the Namorona River. *Anelosimus decaryi* has previously only been known from coastal habitats at low elevations. At Ranomafana (600–800m elevation) we found six colonies containing singleton females and one colony containing a female with an egg case. In contrast to the other species of *Anelosimus* at Ranomafana, *Anelosimus decaryi* colonies did not contain any foreign spiders.

## Supplementary Material

XML Treatment for Anelosimus

XML Treatment for Anelosimus may

XML Treatment for Anelosimus vondrona

XML Treatment for Anelosimus salut

XML Treatment for Anelosimus nazariani

XML Treatment for Anelosimus sallee

XML Treatment for Anelosimus andasibe

XML Treatment for Anelosimus torfi

XML Treatment for Anelosimus hookeri

XML Treatment for Anelosimus lamarcki

XML Treatment for Anelosimus tita

XML Treatment for Anelosimus ata

XML Treatment for Anelosimus darwini

XML Treatment for Anelosimus wallacei

XML Treatment for Anelosimus huxleyi

XML Treatment for Anelosimus buffoni

XML Treatment for Anelosimus moramora

XML Treatment for Anelosimus decaryi

## Supplementary materials

Supplementary material 1 Data matrix Data type: NEXUS file Explanation note: Data matrix set of five aligned and concatenated mtDNA and nuDNA loci. This dataset is made available under the Open Database License (http://opendatacommons.org/licenses/odbl/1.0/). The Open Database License (ODbL) is a license agreement intended to allow users to freely share, modify, and use this Dataset while maintaining this same freedom for others, provided that the original source and author(s) are credited.Ingi Agnarsson, Brian B. Jencik1, Giselle M. Veve, Sahondra Hanitriniaina, Diego Agostini, Seok Ping Goh, Jonathan Pruitt, Matjaž Kuntner
